# Dihydromyricetin Enhances the Chemo-Sensitivity of Nedaplatin via Regulation of the p53/Bcl-2 Pathway in Hepatocellular Carcinoma Cells

**DOI:** 10.1371/journal.pone.0124994

**Published:** 2015-04-27

**Authors:** Lianggui Jiang, Qingyu Zhang, Hao Ren, Sheng Ma, CaiJie Lu, Bin Liu, Jie Liu, Jian Liang, Mingyi Li, Runzhi Zhu

**Affiliations:** 1 Laboratory of Hepatobiliary Surgery, Affiliated Hospital of Guangdong Medical College, Zhanjiang Key Laboratory of Hepatobiliary Diseases, Zhanjiang, China; 2 Department of Gastroenterology, Affiliated Hospital of Guangdong Medical College, Zhanjiang, China; Taipei Medical University, TAIWAN

## Abstract

Chemotherapy is an effective weapon in the battle against cancer. Nedaplatin (NDP) is an improved platinum-containing drug with lower cytotoxicity than other similar drugs. However, the repeated use of NDP results in substantial hepatocyte damage as well as drug resistance in hepatocellular carcinoma (HCC) cases. Therefore, the development of effective chemotherapy strategies that enhance tumor sensitivity to chemotherapeutics and reduce the secondary damage to liver cells is urgently needed. Dihydromyricetin (DHM), a natural flavonoid compound, has been shown to have antitumor activity with no obvious toxicity to normal cells in vitro and in vivo. In this study, DHM and NDP were combined to treat liver cancer cells; we found that DHM functions as a protector of normal cells compared with the use of NDP alone. In addition, the synergy of DHM with NDP enhanced the effect of NDP on the induction of HCC cell apoptosis. We found that the combination caused clear changes in the level of reactive oxygen species (ROS). Furthermore, we demonstrated that the combination of DHM and NDP activated the p53/Bcl-2 signaling pathway, which resulted in mitochondrial dysfunction and induced cell death and growth inhibition in HCC cells.

## Introduction

Platinum drugs significantly attenuate the growth of tumors, but their use is accompanied by the development of chemo-resistance as well as cytotoxicity. Nedaplatin (NDP) is a second-generation platinum drug with broad-spectrum anti-cancer applications. It was first developed by Shionogi & Co Ltd and was approved for sale in Japan in 1995[1]. NDP has been generally used for the treatment of head and neck cancer, esophageal cancer, small cell lung cancer, non-small cell lung cancer, cervical cancer, and ovarian cancer [2–5]. For liver cancer [6], although NDP had fewer side effects than first-generation platinum drugs, it still causes a variety of adverse reactions such as nausea, vomiting and nephrotoxicity [7–9]. We often administer NDP in combination with other chemotherapy drugs such as 5-fluorouracil, paclitaxel, and streptomycin. Synergistic use can produce more pronounced anti-cancer effects [10–13], but multidrug resistance and cell cytotoxicity also develop.

The enhancement of drug sensitivity by combination chemotherapy has been extensively studied. However, few studies have sought to develop a novel compound that protects normal cell functions when combined with platinum drugs.[14–17] Dihydromyricetin is the main active ingredient of flavonoids and has many functions, such as scavenging free radicals as well as anti-oxidation, antithrombotic, antitumor and anti-inflammatory effects. Our lab has demonstrated that DHM is an inducer of apoptosis in HCC cells but a protector of normal liver cells [18]; our previous research has shown that DHM inhibits HCC cell HepG2 by activation of the p53/Bax pathway in vitro. In addition, DHM has an impact on cancer cell migration, proliferation and cell autophagy [19–21]. However, DHM actually has no significant cytotoxicity in normal cells in vitro and in vivo[22]. Based on these findings, we sought to determine whether the combination of NDP and DHM increases the sensitivity of cancer cells to NDP while avoiding obvious injury to normal cells. Here, we perform a prospective study to demonstrate that DHM enhances the curative effects and reduces the damage to normal cells and to elucidate the underlying molecular mechanisms.

## Materials and Methods

### 2.1 Drugs and reagents

DHM was purchased from Sigma-Aldrich and solubilized in dimethylsulfoxide (DMSO) to a final stock concentration of 50 mM and stored at -20°C in the freezer. NDP was purchased from Nanjing East Express Pharmaceutical Corporation and was solubilized in sterilized H_2_O to a final stock concentration of 5 mg/ml and stored at -4°C. Antibodies specific for p53, Bcl-2, Bad, Bax, Bak and GAPDH were purchased from Cell Signaling Technology. Secondary antibodies were obtained from Earthox (Cat: E030120-01).

### 2.2 Cell lines and culture

The SMMC7721 and QGY7701 human hepatocellular carcinoma cell lines and the HL7702 hepatic immortal cell line were kind gifts from Professor Yi Cao (Molecular Pathology Laboratory, Kunming Institution of Zoology, Chinese Academy of Science, Kunming, China). Cells were cultured in RPMI 1640 medium (Gibco, Grand Island, NY) supplemented with 10% heat-inactivated fetal bovine serum (GIBICO, NY), penicillin 100 U/ml and streptomycin 100 U/ml. Cells were maintained in a humidified atmosphere of 95% air with 5% CO_2_ in an incubator at 37°C. These three cell types were grown in standard media, and when the proliferation of the cells was 40%–60%, the cells were treated with different concentrations of DHM and NDP.

### 2.3. Cell morphology assessment

Cells were seeded in 6-well plates with 1.5x10^5^ cells per well. After treatment with DHM and NDP, cells were observed (100×objective) with an inverted microscope (Leica, Germany) and photographed.

### 2.4. Cell inhibition and cytotoxicity assays

MTT preliminary experiments: Cell densities were adjusted to 1×10^4^ cells per 100 μl. Cells were seeded on a 96-well plate with 1×10^4^ cells per well and cultured in an incubator overnight. To select the most effective concentration and time point, QGY7701 and HL7702 cells were treated with 0, 20, 40, 60, 80, 100, 120, 140 and 160 μM DHM for 6, 12 and 24 h. SMMC7721 cells were treated with 0, 25, 50, 75, 100, 125, 150, 175 and 200 μM DHM for 12, 24 and 48 h. The three cell lines (QGY7701, SMMC7721, and HL7702) were pretreated with 0, 2, 4, 6, 8, 10, 12, 14, 16, 18 and 20 μg/ml NDP for 12, 24 and 48 h.

According to the data above, we selected 100 μM DHM combined with 10 μg/ml NDP for the QGY7701 and HL7702 cells; 200 μM DHM was combined with 15 μg/ml NDP for the SMMC7721 cells. After drug exposure, 10 μL MTT solutions (5 mg/ml) were added, and the cells were incubated for 2 hrs. The cell supernatants were carefully removed, and 150 μl DMSO was transferred to each well for resolving of the MTT crystals. The optical density (OD 570 nm) values were recorded by a spectrophotometer.

### 2.5. Colony formation assay

A 0.8% base agar layer and an upper soft agarose (0.7%) layer were pre-coated in 6-well plates in a 37°C incubator for 12 hrs. Cells were pretreated with drugs for 24 hrs. Then, the cells were harvested and plated in each well at 1000 cells per well. After 10 days of culture, the cells were stained with crystal violet, the clones were counted, and cell morphology was photographed.

### 2.6. Apoptosis assay

Briefly, cells were plated in the 6-well plates (1×10^5^ cells per well); 12 h later, the cells were treated with the drugs. After various exposure times, the cells were collected and washed twice with cold D-Hanks buffer solution. Apoptotic cells were quantified using an Annexin V-FITC/PI cell apoptosis detection kit (BD Pharmingen, USA) and monitored using flow cytometry (FACSCalibur, Becton Dickinson). Our experiment strictly complied with the manufacturer’s protocol.

### 2.7. Reactive oxygen species detection

We seeded cells into 6-well plates, which were placed in an incubator overnight to allow for attachment and recovery. Cells were treated with the drugs at different exposure times. Then, we added drugs, and two repeated dosing groups were set up in each group. We set up three controls: a blank control, a positive control (ROSUP), and a DCFH control. We replaced the medium with serum-free medium, and 10 μM DCFH was added per well. After incubation for 20 min in a 37°C incubator, the cells were harvested and analyzed using flow cytometry.

### 2.8. Western blot analysis

Briefly, cells were collected and washed with ice-cold PBS once. The cells were lysed with lysis buffer (10 μl PMSF (100 mM), was added to 1 ml RIPA). The soluble cell lysates were collected after centrifugation at 13000 g for 5 min. Equivalent amounts of protein from each lysate were resolved in 10% SDS–polyacrylamide gels. Bands were transferred to PVDF membranes, which were blocked with 5% skim milk in PBS containing 0.1% Tween 20 (TBST) and then incubated overnight with the specific antibodies at a dilution of 1:1,000 at 4°C. After washing with TBST three times (10 min each), the membranes were incubated at room temperature for 2 hrs with the secondary antibody at a dilution of 1:2,000. Chemiluminescent substrate (ECL, GE Healthcare) was added to the membranes, which were photographed using a protein analysis system (ProteinSimple, USA).

### 2.9. Mitochondrial staining

Mito-tracker green was purchased from Beyotime Corporation (Shanghai, China), and the cells were incubated with mito-tracker green probes for 30 min. Once the mitochondria were labeled, the cells were fixed with 4% paraformaldehyde, and the cell nuclei were stained with DAPI for 5 min. Detailed procedures were performed according to the manufacturer’s protocol.

### 2.10. Statistical analysis

The statistical analyses were performed using GraphPad Prism 5 software. All results are represented as the mean ± SD. Significant differences were evaluated using Student’s t-test and were considered significant at the * P<0.05, ** P<0.01 or *** P<0.001 level. All data in the figures shown in this article were obtained from at least three independent experiments.

## Results

### 3.1 The combination of DHM and NDP showed a synergistic effect on the inhibition of tumor cell proliferation but no significant impact on non-tumor cells

**Fig 1:**
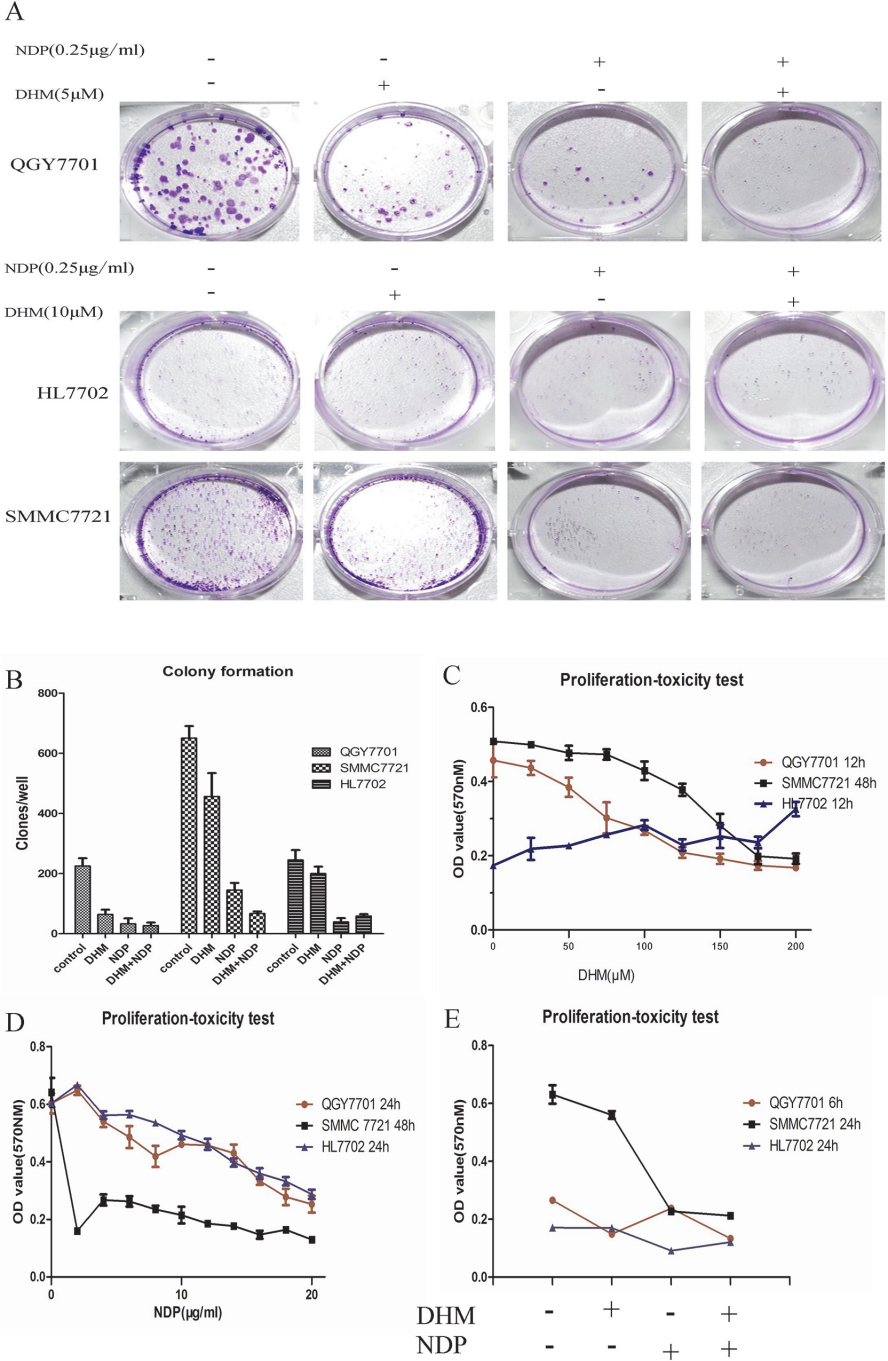
DHM combination with NDP inhibits the ability of colony formation of liver cancer cells. (A) Colony formation capability assay with different treatments of DHM and NDP in HCC cells. The images were captured with a Nikon camera (Japan). (B) The clones were quantified and presented as a statistical figure. (C, D) The proliferation-toxicity of NDP or DHM in QGY7701, HL7702 and SMMC7721 cells with various drug concentrations and treatment durations were assayed using the MTT method (means ± S.D). (D) Proliferation and toxicity of different drug combinations were measured using the MTT assay (means ± S.D). Each experiment was repeated at least three times.

For the hepatoma cells, the effect of the combination of NDP and DHM showed a greater effect in the inhibition of colony formation ability than the use of DHM or NDP individually. In normal liver cells, DHM possesses functions in preserving cell colony formation capability and reducing the inhibitory effect induced by NDP (Fig 1-A and 1-B). The three cells lines showed a time-concentration dependence on NDP, and we selected the 10 μg/ml concentration for the subsequent experiments in the QGY7701 and HL7702 cells. For the SMMC7721 cells, a 15 μg/ml concentration of NDP was adopted for the subsequent experiments (Fig 1-C). The QGY7701 and SMMC7721 cell proliferation showed a time/concentration-dependence on DHM, but this phenomenon was not observed in the HL7702 cells. We selected a sensitive concentration for the QGY7701 and HL7702 cells of 100 μM DHM and a concentration for the SMMC7721 cells of 200 μM DHM (Fig 1-D). We found that the DHM combination with NDP showed a synergy effect to inhibit cancer cell proliferation. Interestingly, DHM reduced the inhibition of proliferation induced by NDP treatment in the HL7702 cells. (Fig 1-E)

### 3.2 DHM enhanced NDP–induced tumor cell apoptosis but protected non-tumor cells from cell apoptosis

**Fig 2:**
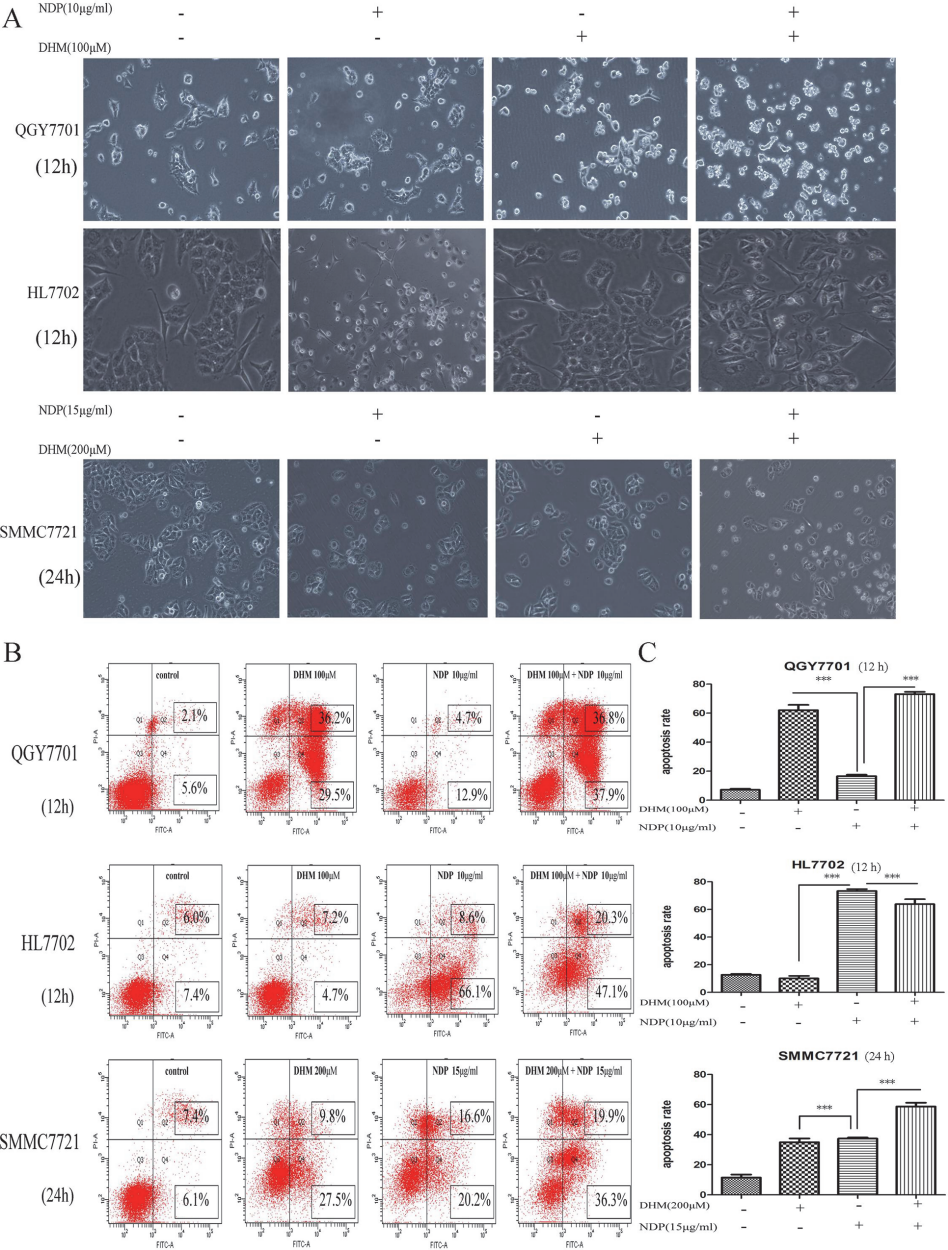
Synergistic effect of DHM and NDP promotes liver cancer cell apoptosis. (A) The effect of each drug individually and in combination on cell apoptosis in QGY7701, HL7702 and SMMC7721 cells with different concentrations and treatment durations were monitored under microscopy (100×). (B, C) The apoptosis of QGY7701, HL7702 and SMMC7721 cells induced by the drugs individually and in combination at different concentrations and treatment durations were detected using FITC Annexin V-PI Apoptosis detection kit (BD Pharmingen, USA) and measured by flow cytometry analysis (means ± S.D). Each experiment was repeated at least three times.

The combination of DHM with NDP produced a clear synergistic effect on the promotion of apoptosis in the QGY7701 and SMMC7721 cells. The cell morphology showed obvious changes, which were photographed with the microscope. Remarkably, DHM exhibited an obvious protective effect on the HL7702 cells and significantly reduced cell apoptosis induced by NDP treatment (Fig 2-A). Cell apoptosis was detected by FITC Annexin V-PI Apoptosis detection kit (BD Pharmingen, USA) and measured using flow cytometry (Fig 2-B). Cells apoptosis ratios of drug treatment were calculated and presented as a statistical figure (Fig 2-C). The data showed that DHM combined with NDP increasingly induced cell apoptosis in hepatic cancer cells but reduce the side-effect to the normal cells.

### 3.3 DHM enhanced the activation of the p53/Bcl-2 signaling pathway induced by NDP treatment

**Fig 3:**
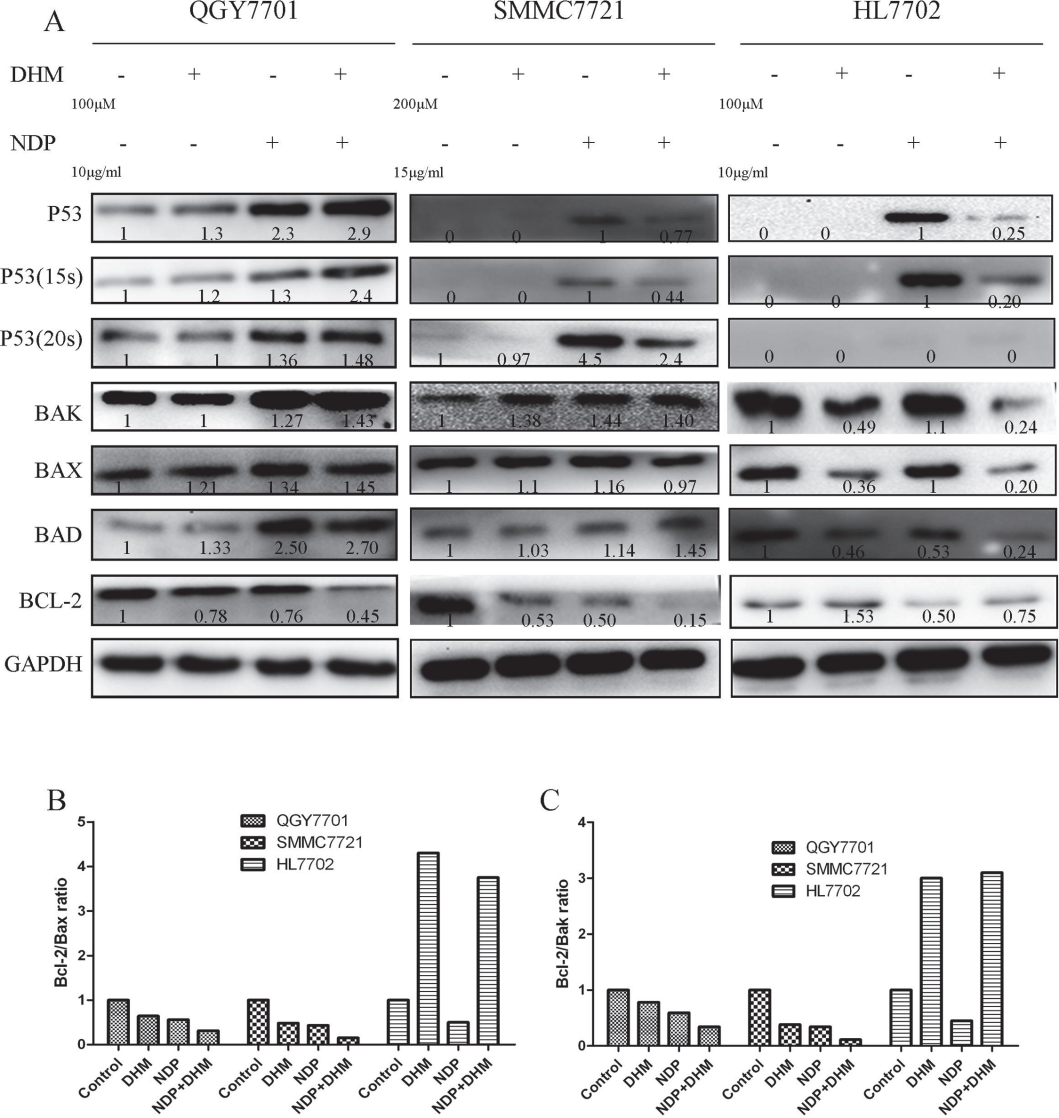
Apoptotic signaling pathway protein expression was evaluated using western blotting. (A) Apoptotic-related protein expression after drug exposure in QGY7701, HL7702 and SMMC7721 cells was quantitated by western blot. (B, C) The Bcl-2/Bax and Bcl-2/Bak protein ratios were measured by optical analysis with ImageJ software.

The protein levels of Bak, Bax and Bad were clearly enhanced in response to the combination of NDP and DHM in the QGY7701 and SMMC7721 cells. In parallel, the combination produced an inhibitory effect on the Bcl-2 protein levels. In the HL7702 cells, the opposite trend was observed. In addition, DHM combined with NDP promoted the activation of p53 and the phosphorylation of the 15-serine of p53 in the QGY7701 and SMMC7721 cells. However, these effects induced by NDP in tumor cells were rescued by DHM in the non-tumor HL7702 cells. (Fig 3-A). Furthermore, the interaction of Bcl-2 with Bax and Bak modulates mitochondrial functions and determines the cell fate of tumors. A higher ratio of Bcl-2/Bax or Bak always results in cell apoptosis. Our study found that the Bcl-2/Bax or Bcl-2/Bak ratios were downregulated in QGY7701 and SMMC7721 cells but were upregulated in HL7702 cells by the DHM and NDP combination (Fig 3-B and 3-C).These results indicated that DHM could promote NDP-induced hepatoma cells apoptosis via activation of the p53/Bcl-2 pathways and relieve the NDP induced damage by maintaining the Bcl-2/Bax or Bcl-2/Bak balance in normal liver cells.

### 3.4 DHM inhibited ROS production in the HL7702 and SMMC7721 cells but had no effect on the ROS level in the QGY7701 cells after NDP treatment

**Fig 4:**
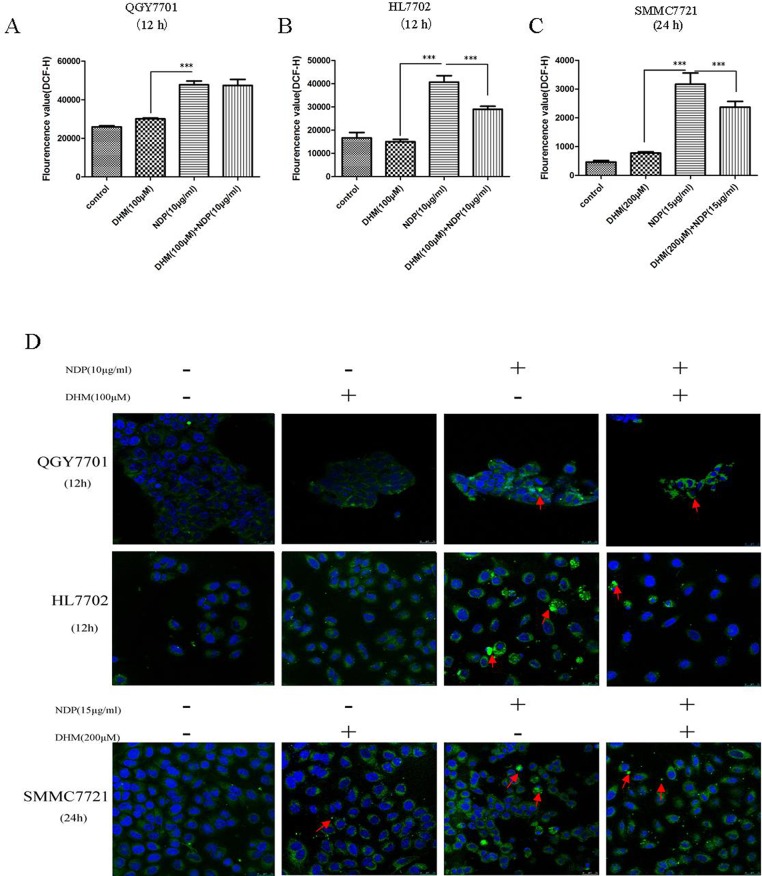
The alteration of ROS in normal liver cells and hepatoma cells. (A,B,C) The level of ROS changes in QGY7701, HL7702 and SMMC7721 cells after treatment with DHM and NDP were detected using the DCFH assay. (B) The morphological changes in the mitochondria in QGY7701, HL7702 and SMMC7721 cells after treatment with the drug individually and in combination were detected using mito-tracker green. Each experiment was repeated at least three times.

ROS play a crucial role in chemotherapy for cancer prevention. Higher levels of ROS damage DNA and induce cell apoptosis. The combination of the two drugs showed no elevation in ROS compared to the use of NDP alone. Notably, DHM reduced the ROS level induced by NDP treatment in the Smmc7721 and HL7702 cells (Fig 4-A, 4-B and 4-C). DHM reduced the ROS level but the ROS levels in the tumor cells were still higher than that in the non-tumor cells. Therefore, ROS production and the balance of ROS in cells play an important role in cell fate determination. DHM regulates the ROS production with NDP treatment in different cells and determinates different cell fate.

### 3.5 DHM attenuated mitochondria damage resulting from NDP treatment in the HL7702 cells but did not have a positive effect on the QGY7701 and SMMC7721 HCC cells

**Fig 5:**
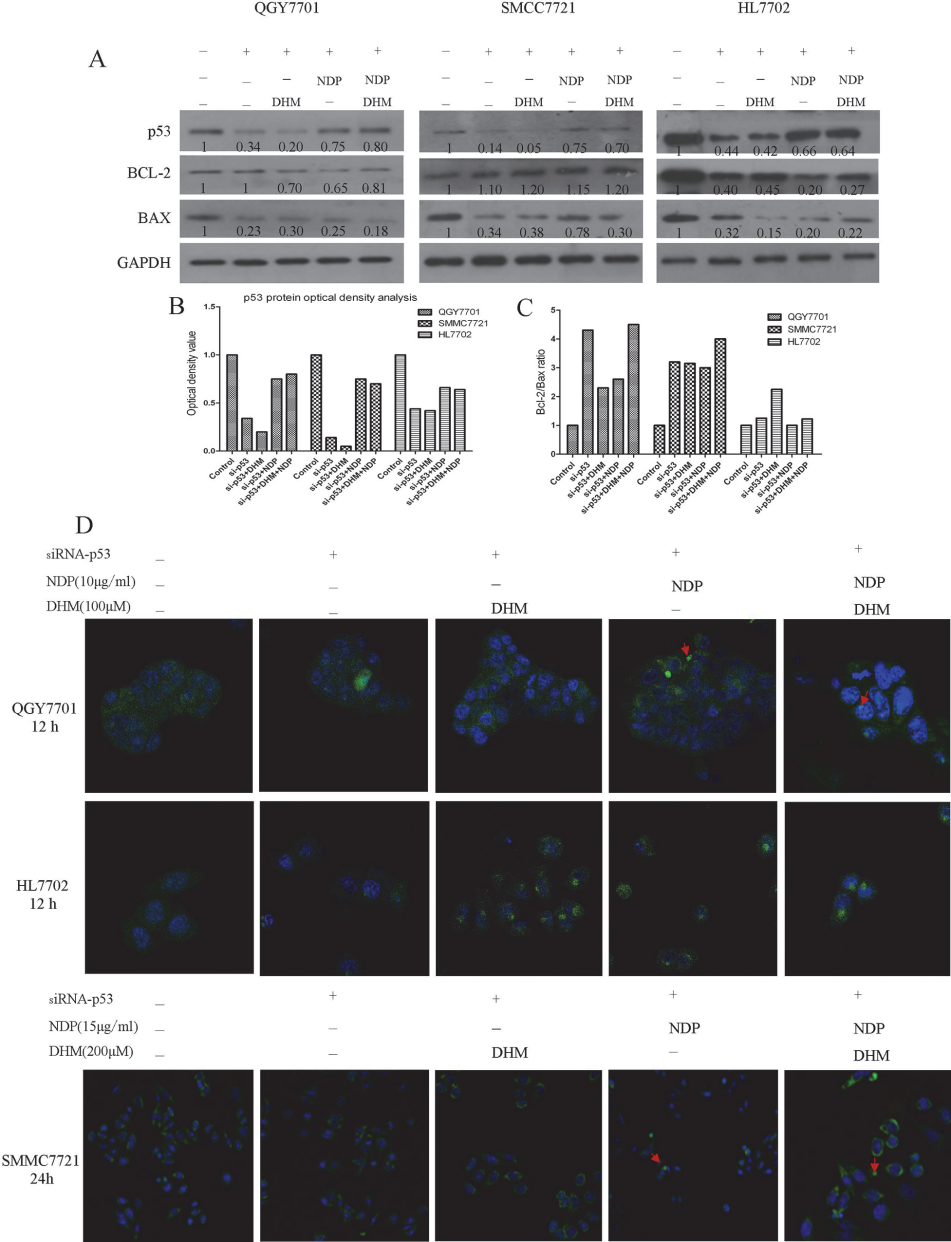
p53/Bcl-2 pathway was implicated in DHM and NDP induced dysfunction of mitochondria. (A, B) p53-siRNA was used to knockdown of p53 and rescued the upregulation of p53 in hepatoma cells after DHM and NDP treatment. (C) Knockdown of p53 inhibited the downregulation of Bcl-2/Bax ratio caused by DHM and NDP treatment. (D) After si-RNA interference was performed, the morphological alteration of mitochondria in QGY7701, HL7702 and SMMC7721 cells after treatment with the drug individually and in combination were detected by using mito-tracker green.

Many chemotherapeutic agents induce tumor cell apoptosis by affecting mitochondrial function. Furthermore, disruption of Bcl-2/Bax and Bcl-2/Bak always results in dysfunction of mitochondria. We sought to determine whether the synergistic effect of DHM and NDP resulted from mitochondrial dysfunction. We stained the mitochondria and found that the morphological changes induced by NDP and DHM treatment, and the combination of the two drugs synergetically increased the numbers of abnormal cells (Fig 4-D). To demonstrate the role of p53/Bcl-2 pathway in the synergetic effect of DHM with NDP in deregulation of mitochondria, we design si-RNA(JiMa, Shanghai, China) specific to p53 mRNA for down-regulation of p53 expression. p53 si-RNA transfection rescued DHM and NDP combined administration induced p53 upregulation and the downregulation of Bcl-2/Bax ratio in QGY7701,SMMC7721 and HL7702 cells(Fig 5-A, 5-B and 5-C). Cells were stained mito-tracker green, and the results indicated that knockdown of p53 remarkably reduced the damaged mitochondria cause by DHM and NDP treatment (Fig 5-D). We conclude that DHM affected the function of mitochondria by modulating the balance of Bcl-2/Bax and Bcl-2/Bak ratio via p53/Bcl-2 pathway.

## Discussion

Natural products provide an enormous pool for the development of tumor chemotherapeutics, such as taxol and vinblastine. Flavonoids are a type of active components that are widely distributed in plants. Many studies have demonstrated that flavonoids have functions in cancer prevention/development. DHM, a flavonoid component from hovenia, has many effects, including antioxidant, anti-tumor and anti-alcohol intoxication activities. Our team discovered that DHM promotes HCC regression through the activation of the p53-dependent cell apoptosis pathway. Intriguingly, we also found that DHM was not cytotoxic in mice and actually protects non-tumor cells from NDP-induced cell injury. Therefore, we sought to determine whether DHM could be combined with a clinical drug to relieve the side effects that affect normal tissues and to enhance the sensitivity of tumor cells.

To confirm the appropriate concentration, we first performed an MTT assay to determine the most effective concentration and time treatment of the drugs. DHM inhibited the HCC cell viability in a significant time- and dose-dependent manner, whereas DHM seems to also have a proliferation-promoting effect. NDP showed cytotoxicity to both tumor cells and non-tumor cells. We selected the DHM and NDP concentrations according to the IC50 after each drug was administered to tumor cells. We sought to determine whether the combination of the two drugs enhances the sensitivity of tumor cells.

The results showed that the combination of NDP and DHM induced more significant apoptosis in the tumor cells but had almost no impact on the non-tumor cells. The Western blot results showed that the p53 and p-p53 (15ser) levels were increased in the QGY7701 and SMMC7721 cells. In parallel, the Bcl-2 family proteins presented as Bax and Bak were elevated but Bcl-2 was reduced after administration of the DHM NDP combination in tumor cells. In the non-tumor cells (HL7702), DHM reversed the p53 pathway activation and maintained the balance of the Bcl-2 family members. Because the aberrant p53/Bcl-2 pathway was closely related to mitochondria-induced cell apoptosis, we proposed that the DHM combination with NDP impacts mitochondrial functions and results in different cell fates. We evaluated the mitochondrial function by tracking the cellular localization and morphology of the mitochondria after NDP and DHM application. We found that the mitochondria showed abnormal changes in tumor cells that may result from elevation of the Bax/Bcl-2 and Bak/Bcl-2 ratios, which were involved in modulating mitochondrial function. In addition, we demonstrated the p53/Bcl-2 pathway was involved in the regulation of mitochondria functions by si-RNA interference. The results also showed that NDP significantly increased the ROS level but that DHM rescued the ROS level elevation provoked by NDP stimulation. It is not known why the same combination of the two drugs resulted in different cell events; however, an explanation is that the tumor cells were not healthy due to the mitochondria being affected by NDP exposure, and it is difficult for DHM to mediate mitochondrial recovery after mitochondrial damage. It is still unknown why DHM alone also induces cell death in tumor cells. We think that DHM is a reducibility compound that inhibits the ROS production that is necessary for tumor cell growth and returns the ROS levels to a normal level.

In conclusion, we found that the DHM combination promoted the NDP chemotherapeutic sensitivity of liver cancer cells and also reduced the cytotoxicity to normal liver cells in vitro. We discovered that DHM application played a role in the regulation of the p53/Bcl-2 pathway, impacted the function of mitochondria and modulated ROS production. Although a prospective target is that DHM could regulate the ROS production induced by NDP treatment and promote the ROS-induced tumor cells apoptosis with less side-effect to the normal cells of patients, in vivo studies should be performed to test the advantages of the DHM and NDP combination. We believe that DHM is a potential compound that will be clinically effective for tumor prevention.

## Supporting Information

S1 Fig Combination of DHM and NDP inhibits the colony formation ability of QGY7701 cells. Colony formation ability was measured by plate colony formation experiment.
(TIF)

S2 Fig Combination of DHM and NDP inhibits the colony formation ability of SMMC7721 cells. Colony formation ability was measured by plate colony formation experiment.
(TIF)

S3 Fig Combination of DHM attenuates impairing of colony formation ability resulted from NDP treatment in HL7702 cells. Colony formation ability was measured by plate colony formation experiment.
(TIF)

S4 Fig Combination of DHM and NDP affect the colony formation ability of three cell lines (QGY7701, SMMC7721, and HL7702). Colony numbers were counted and presented as a statistical figure.
(TIF)

S5 Fig NDP decreased the cell viability of three cell lines (QGY7701, SMMC7721, and HL7702). Cells were treated with various concentrations NDP and the cell viability was measured by MTT.
(TIF)

S6 Fig DHM decreased the cell viability of hepatoma cell lines (QGY7701, SMMC7721) and affects weakly to non-hepatoma cell HL7702. Cells were treated with various concentrations NDP and the cell viability was measured by MTT.
(TIF)

S7 Fig Combination of DHM with NDP inhibited the cell viability of hepatoma cell lines (QGY7701, SMMC7721) and reduced the NDP treatment mediated cell viability inhibition in HL7702 cells. Cells were treated with various concentrations NDP and the cell viability was measured by MTT.
(TIF)

S8 Fig QGY7701cells were treated with NDP and DHM separately and combination. The cell morphology was monitored by a Leica inverted microscope.
(TIF)

S9 Fig HL7702 cells were treated with NDP and DHM separately and combination. The cell morphology was monitored by a Leica inverted microscope.
(TIF)

S10 Fig SMMC7721 Cells were treated with NDP and DHM separately and combination. The cell morphology was monitored by a Leica inverted microscope.
(TIF)

S11 Fig The apoptosis of QGY7701 cells induced by the DHM and NDP individually and combination at different concentrations and treatment durations. The apoptosis of cells were measured by flow cytometry analysis.
(TIF)

S12 Fig The apoptosis of HL7702 cells induced by the DHM and NDP individually and combination at different concentrations and treatment durations. The apoptosis of cells were measured by flow cytometry analysis.
(TIF)

S13 Fig The apoptosis of SMMC7721 cells induced by the DHM and NDP individually and combination at different concentrations and treatment durations. The apoptosis of were measured by flow cytometry analysis.
(TIF)

S14 Fig A statistical figure for apoptosis rate induced by DHM and NDP synergic or individual treatment in three cell lines. (TIF)

S15 Fig Combination of DHM with NDP activated the p53/Bcl-2 pathway in QGY7701 cells. The apoptotic proteins were detected by western blot in QGY7701 cells.
(TIF)

S16 Fig Combination of DHM with NDP attenuated the activation of p53/Bcl-2 pathway in HL7702 cells. The apoptotic proteins were detected by western blot in HL7702 cells.
(TIF)

S17 Fig Combination of DHM with NDP activated the p53/Bcl-2 pathway in SMMC7721 cells. The apoptotic proteins were detected by western blot in SMMC7721 cells.
(TIF)

S18 Fig DHM reduced the ROS level increased by NDP treatment in three cell lines. Reactive oxygen species were detected by using the DCFH assay in three cell lines (QGY7701, SMMC7721, and HL7702).
(TIF)

S19 Fig Combination of DHM with NDP affected the mitochondria morphology in QGY7701 cells. Mitochondria morphology was evaluated by mito-tracker green staining after drugs treatment in QGY7701 cells.
(TIF)

S20 Fig DHM reduced the mitochondria morphology damage caused by NDP treatment in HL7702 cells. Mitochondria morphology was evaluated by mito-tracker green staining after drugs treatment in HL7702 cells.
(TIF)

S21 Fig Combination of DHM with NDP affected the mitochondria morphology in SMMC7721 cells. Mitochondria morphology was evaluated by mito-tracker green staining after drugs treatment in SMMC7721 cells.
(TIF)

S22 Fig Knockdown p53 relieved the p53/Bcl-2 pathway activation in QGY7701 cells. The apoptotic proteins were detected by western blot after p53 was knockdown in QGY7701 cells.
(TIF)

S23 Fig Knockdown p53 relieved the p53/Bcl-2 pathway activation in SMMC7721 cells. The apoptotic proteins were detected by western blot after p53 was knockdown in SMMC7721 cells.
(TIF)

S24 Fig Knockdown p53 relieved the p53/Bcl-2 pathway activation in HL7702 cells. The apoptotic proteins were detected by western blot after p53 was knockdown in HL7702 cells.
(TIF)

S25 Fig Protein content was evaluated by optical density analysis using ImageJ software. (TIF)

S26 Fig Knockdown p53 relieved the p53/Bcl-2 pathway activation. Bcl-2/Bax ratio were calculated using optical density value
(TIF)

S27 Fig Knockdown p53 attenuated the NDP/DHM treatment caused mitochondria morphology injury in QGY7701 cells. Mitochondria morphology was evaluated by mito-tracker green staining after drugs treatment in QGY7701while p53 was knockdown by siRNA transfection.
(TIF)

S28 Fig Knockdown p53 attenuated the NDP/DHM treatment caused mitochondria morphology injury in HL7702 cells. Mitochondria morphology was evaluated by mito-tracker green staining after drugs treatment in HL7702 while p53 was knockdown by siRNA transfection.
(TIF)

S29 Fig Knockdown p53 attenuated the NDP/DHM treatment caused mitochondria morphology injury in SMMC7721 cells. Mitochondria morphology was evaluated by mito-tracker green staining after drugs treatment in SMMC7721 while p53 was knockdown by siRNA transfection.
(TIF)
