# Supplementary figures and images for: Dihydromyricetin Enhances the Chemo-Sensitivity of Nedaplatin via Regulation of the p53/Bcl-2 Pathway in Hepatocellular Carcinoma Cells
Source: PLoS One. 2015 Apr 27;10(4):e0124994. doi: 10.1371/journal.pone.0124994 (PMC4411137; doi:10.1371/journal.pone.0124994)

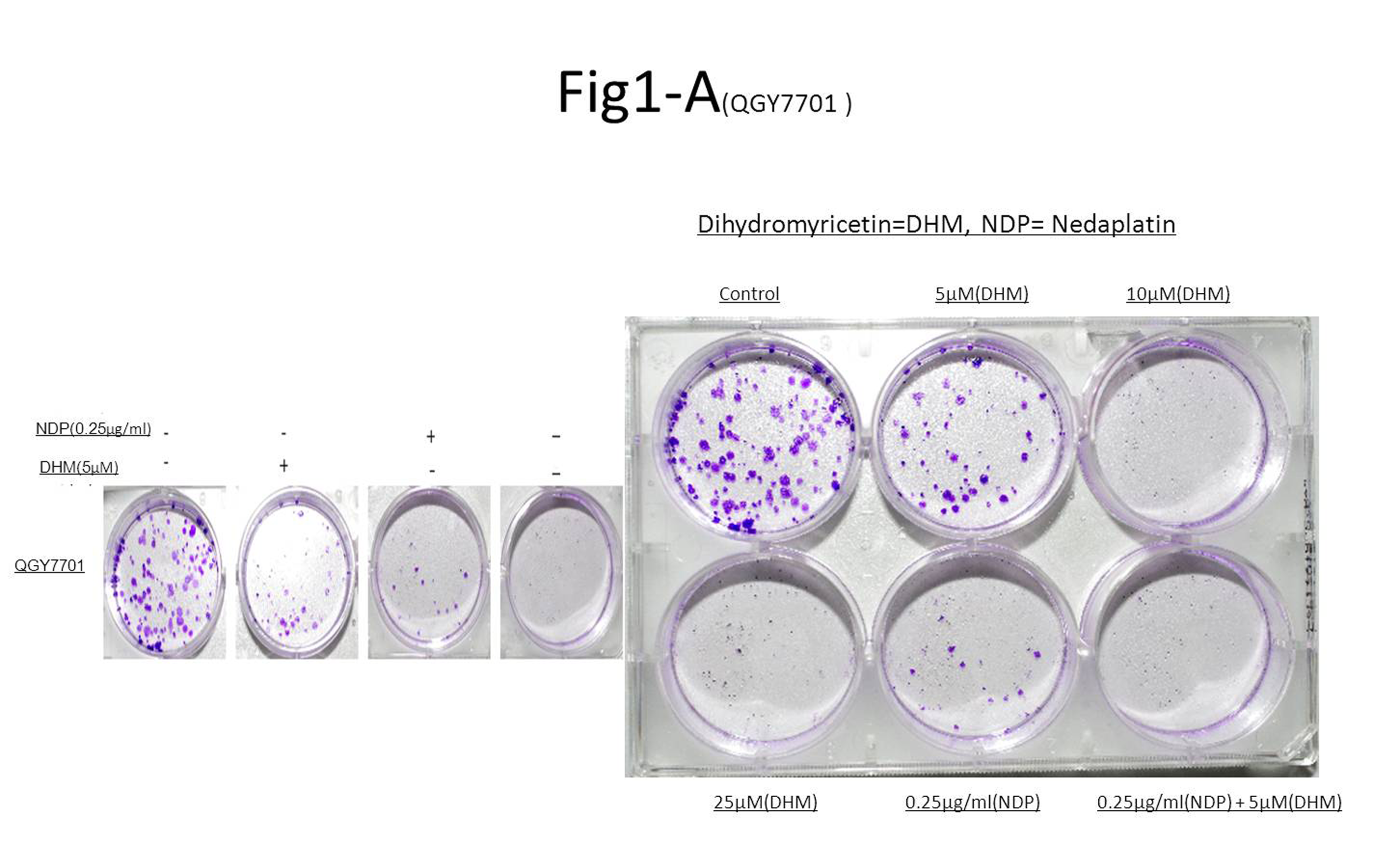

Supplement: S1 Fig — Colony formation ability was measured by plate colony formation experiment. (TIF) [file pone.0124994.s001.tif]

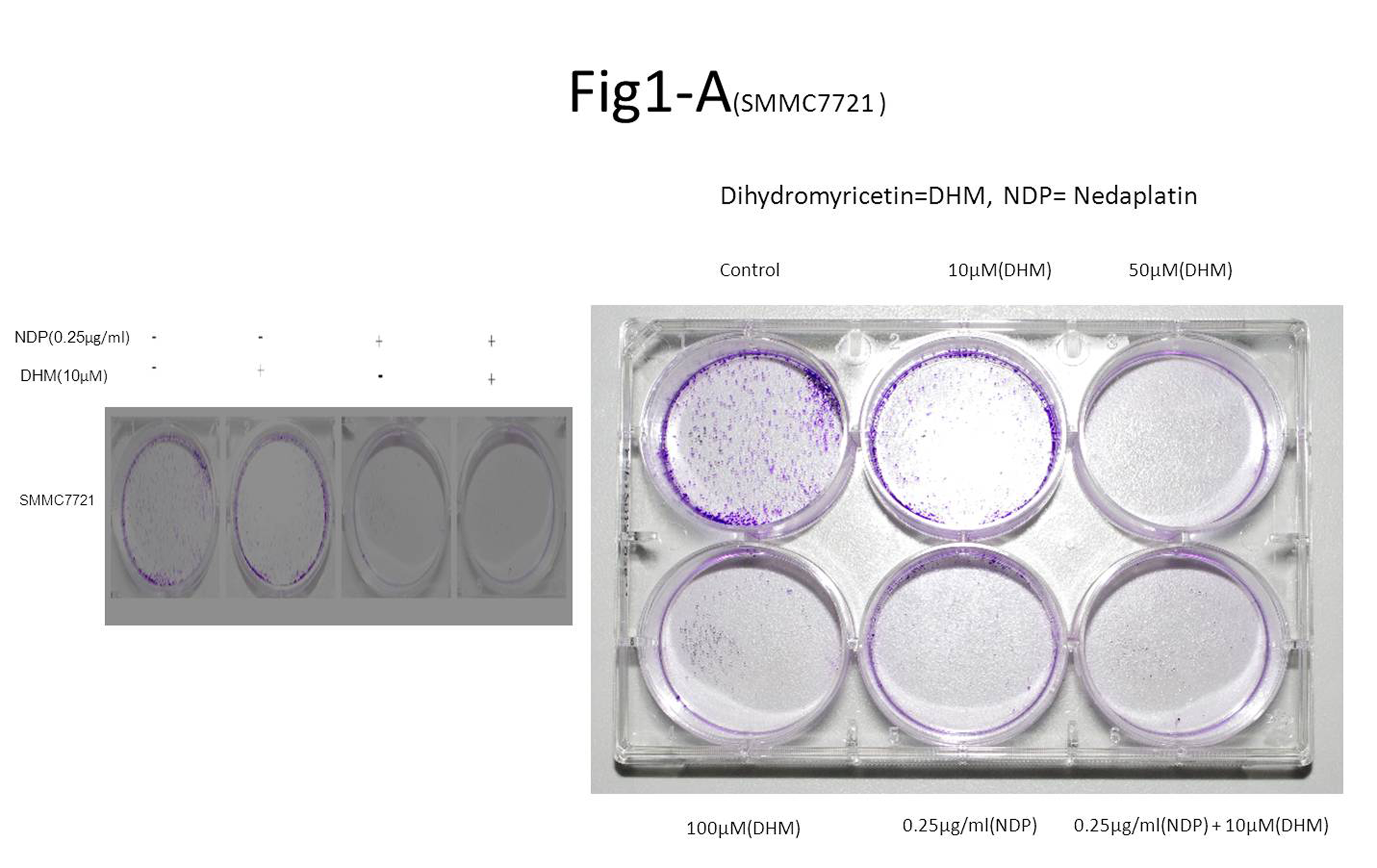

Supplement: S2 Fig — Colony formation ability was measured by plate colony formation experiment. (TIF) [file pone.0124994.s002.tif]

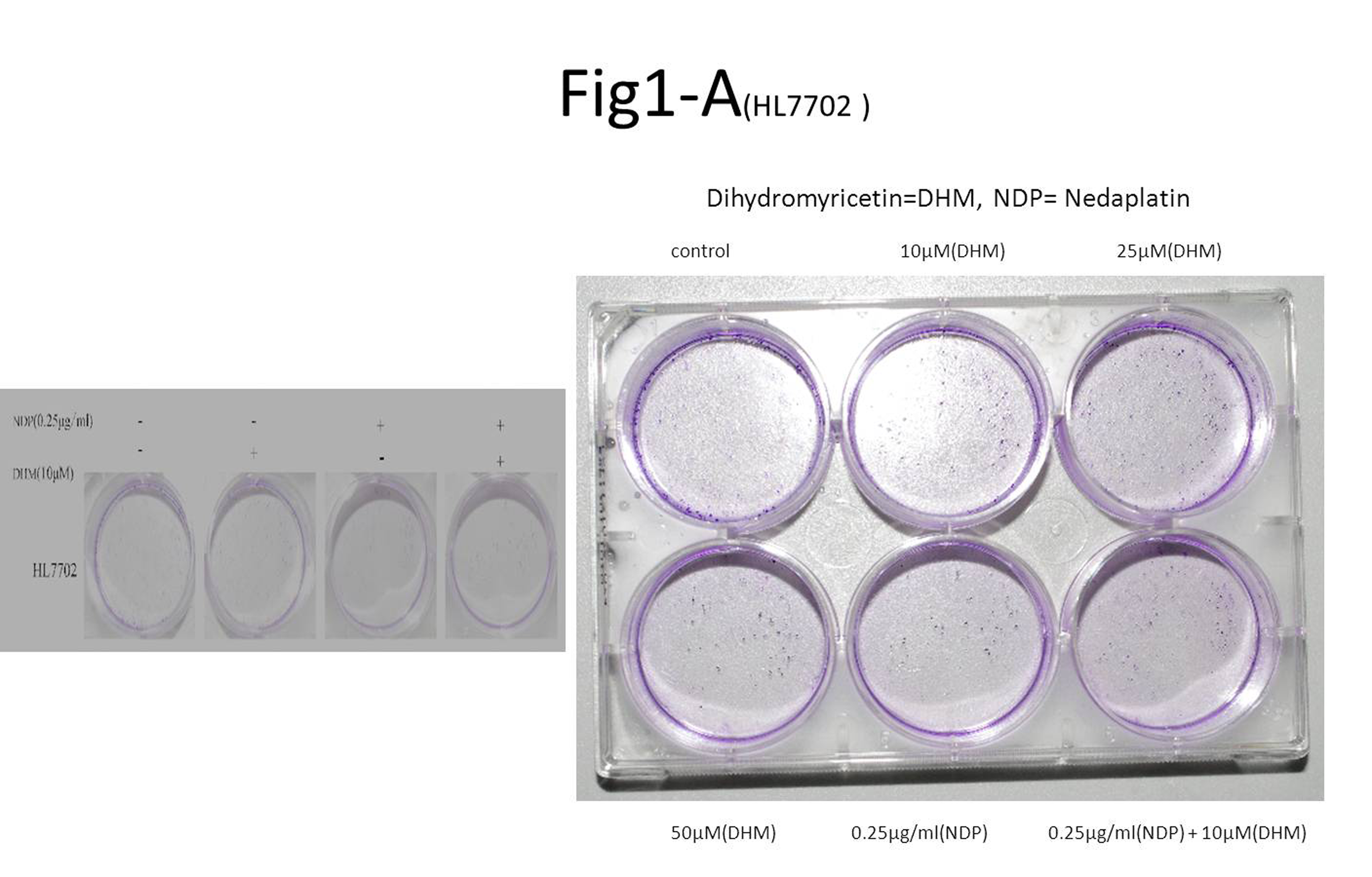

Supplement: S3 Fig — Colony formation ability was measured by plate colony formation experiment. (TIF) [file pone.0124994.s003.tif]

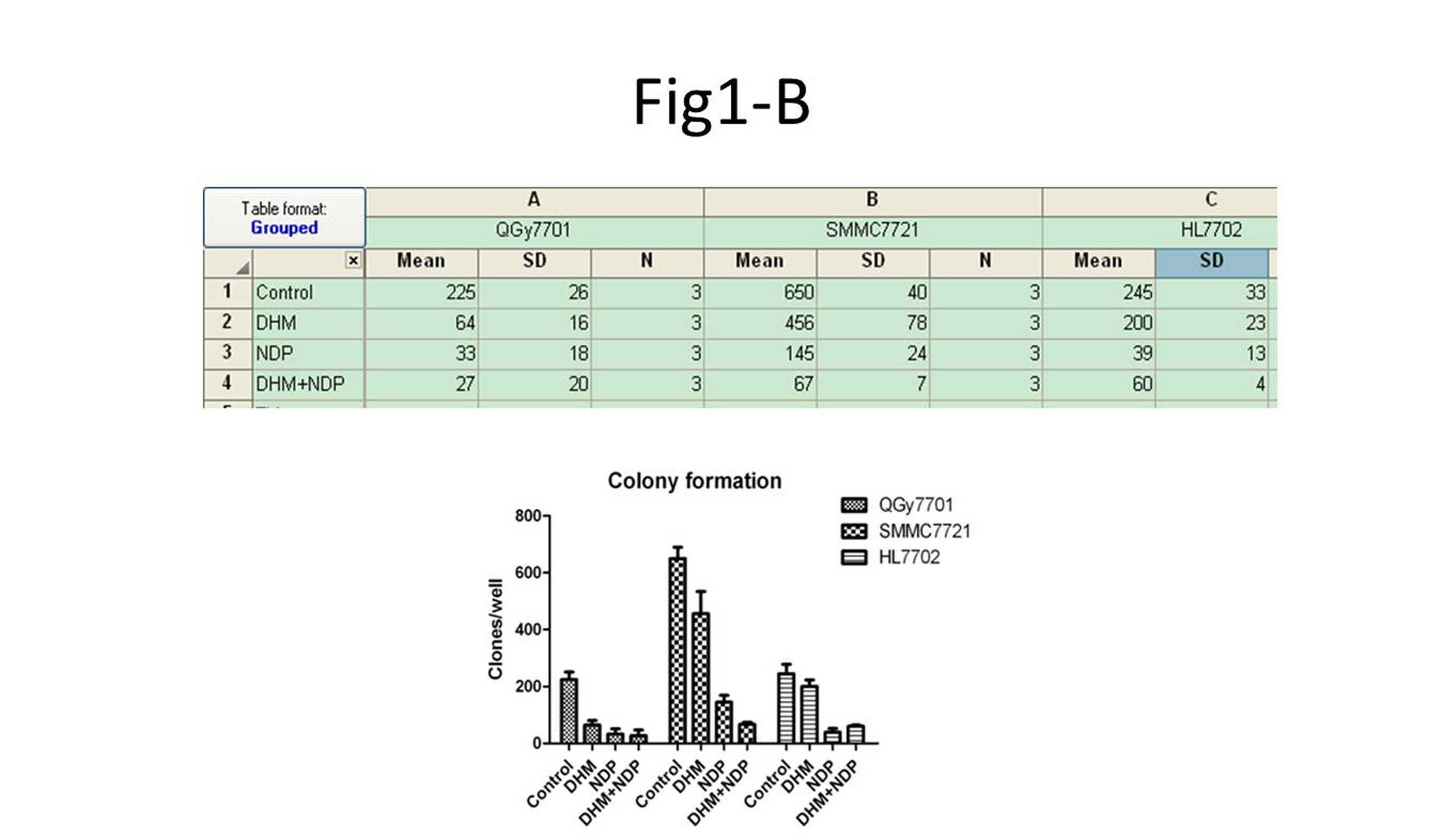

Supplement: S4 Fig — Colony numbers were counted and presented as a statistical figure. (TIF) [file pone.0124994.s004.tif]

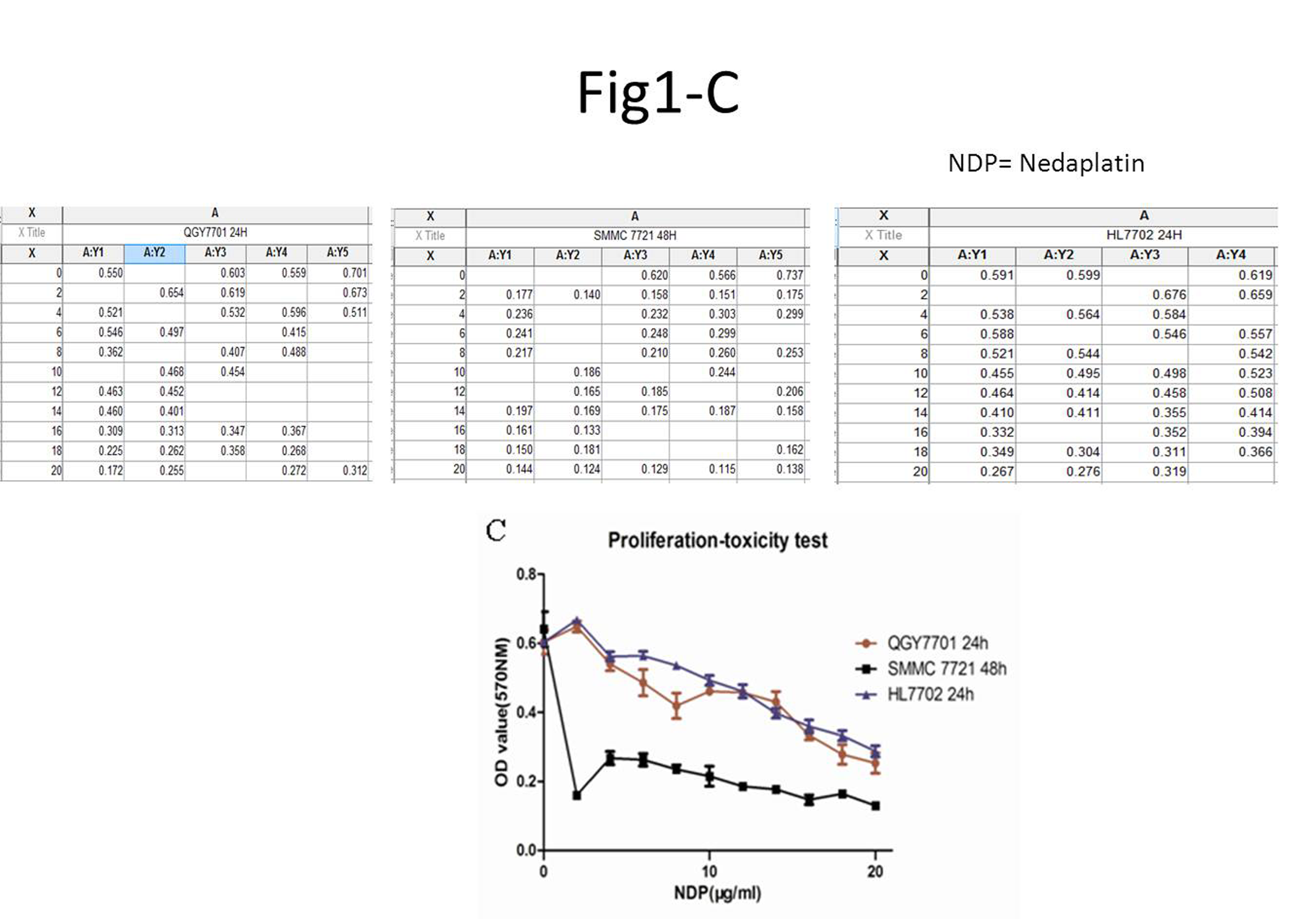

Supplement: S5 Fig — Cells were treated with various concentrations NDP and the cell viability was measured by MTT. (TIF) [file pone.0124994.s005.tif]

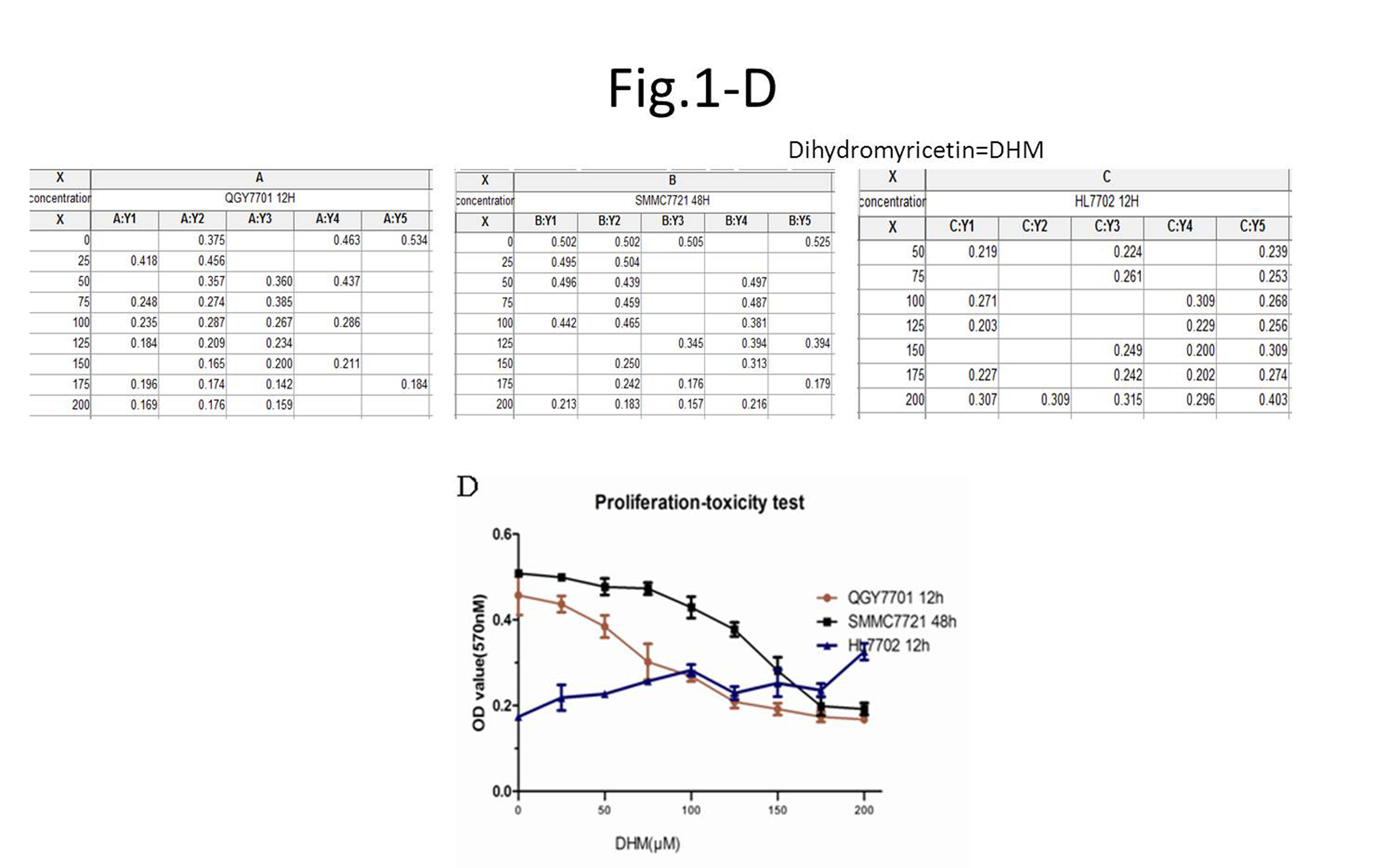

Supplement: S6 Fig — Cells were treated with various concentrations NDP and the cell viability was measured by MTT. (TIF) [file pone.0124994.s006.tif]

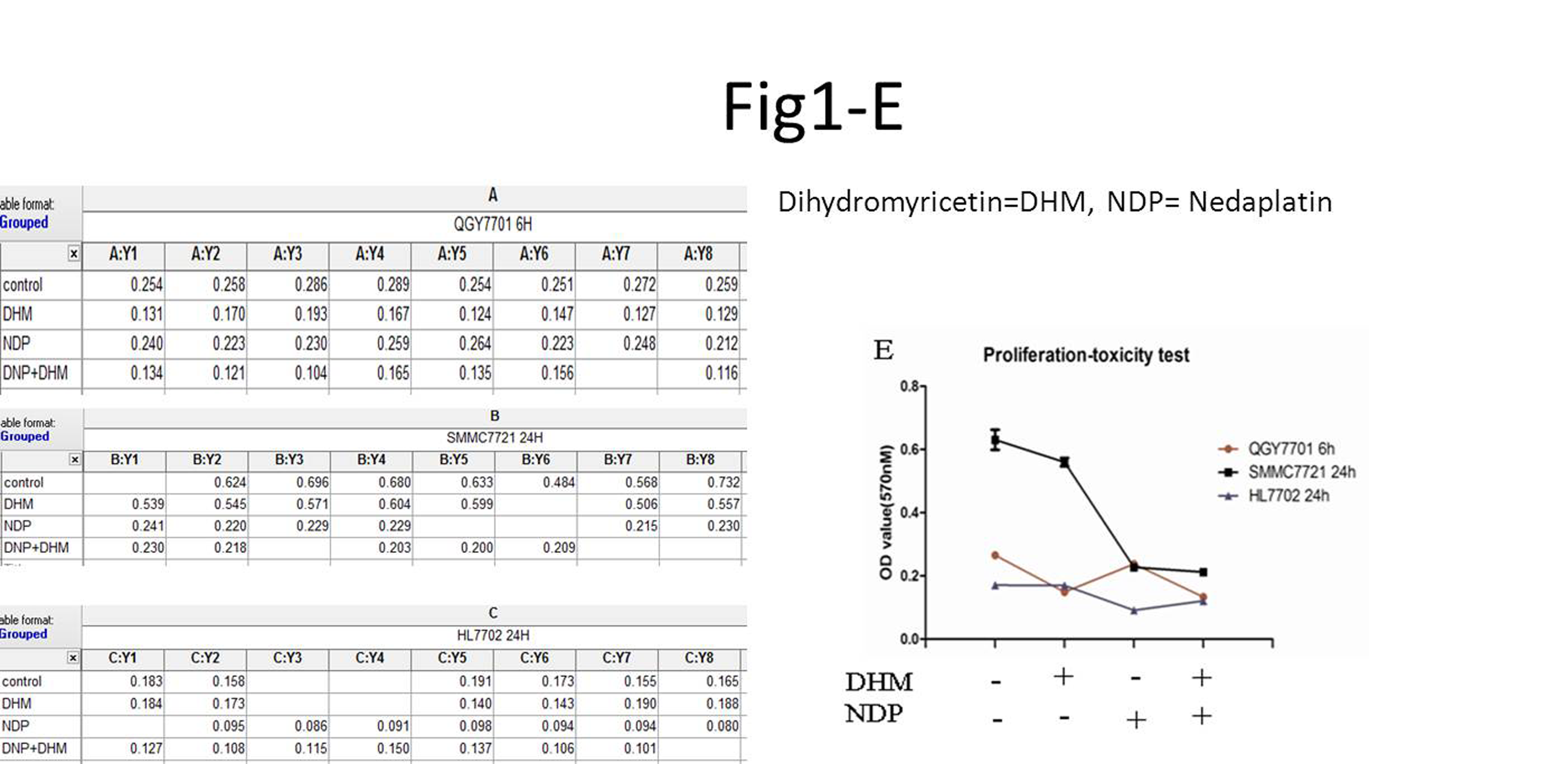

Supplement: S7 Fig — Cells were treated with various concentrations NDP and the cell viability was measured by MTT. (TIF) [file pone.0124994.s007.tif]

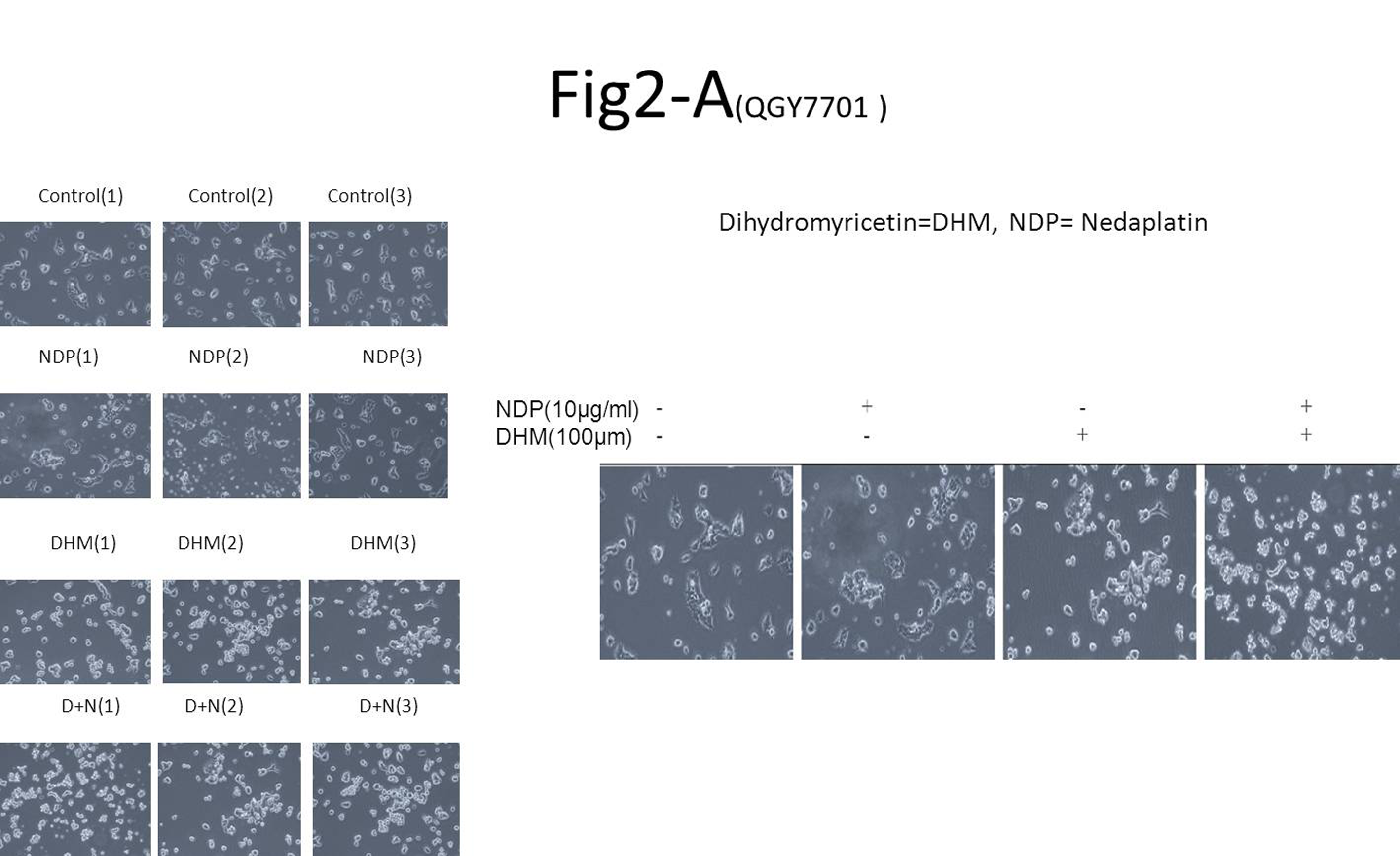

Supplement: S8 Fig — The cell morphology was monitored by a Leica inverted microscope. (TIF) [file pone.0124994.s008.tif]

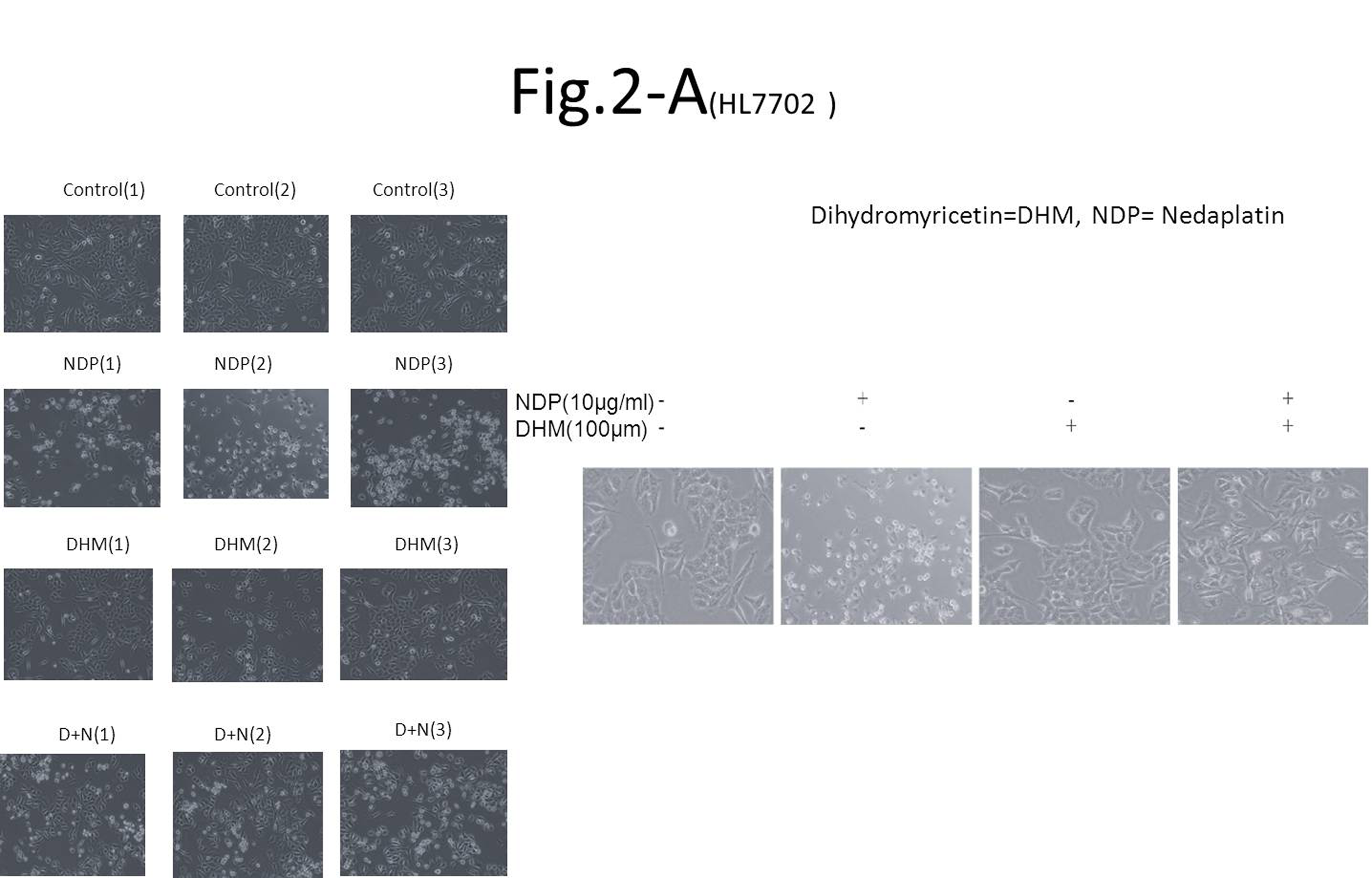

Supplement: S9 Fig — The cell morphology was monitored by a Leica inverted microscope. (TIF) [file pone.0124994.s009.tif]

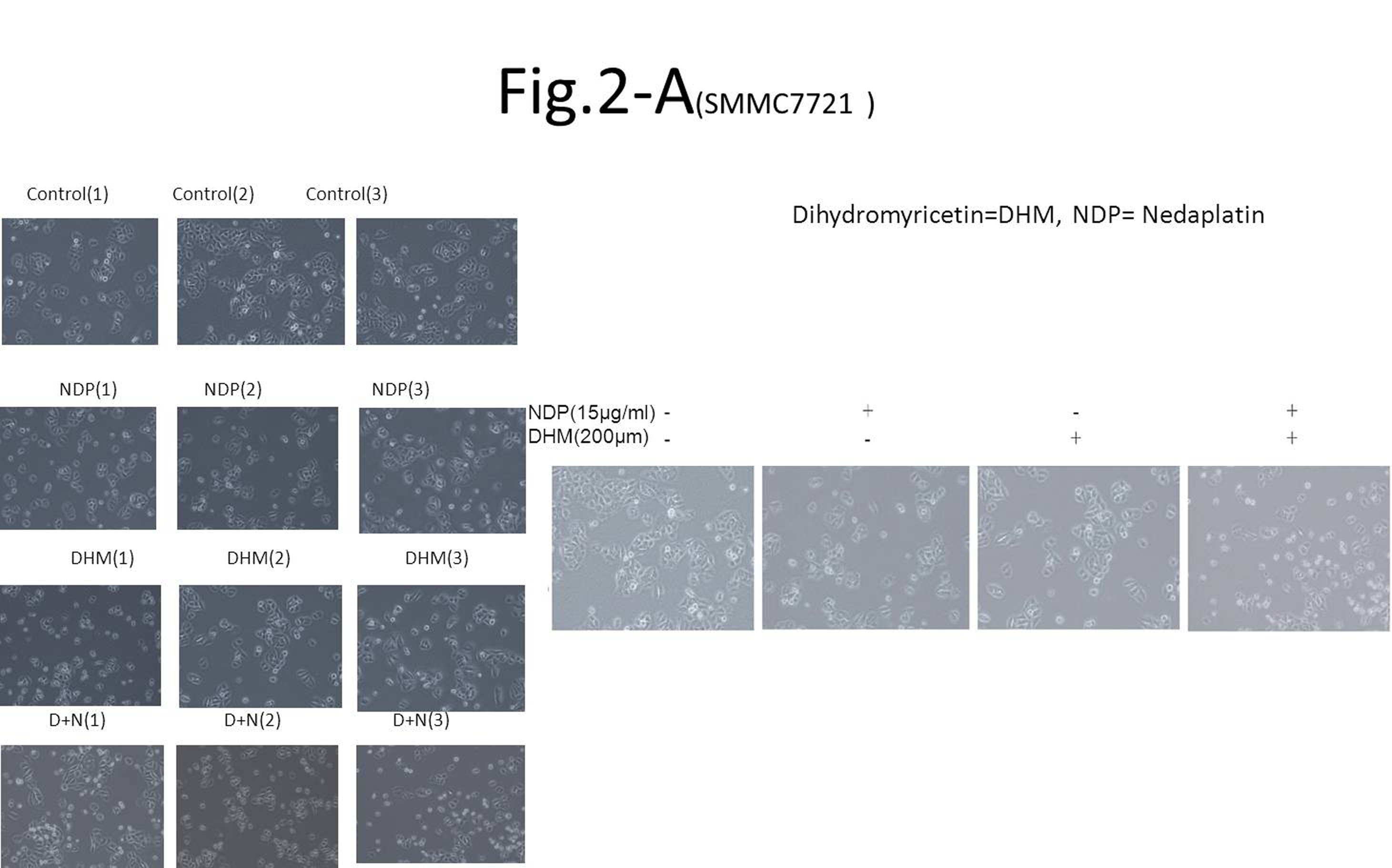

Supplement: S10 Fig — The cell morphology was monitored by a Leica inverted microscope. (TIF) [file pone.0124994.s010.tif]

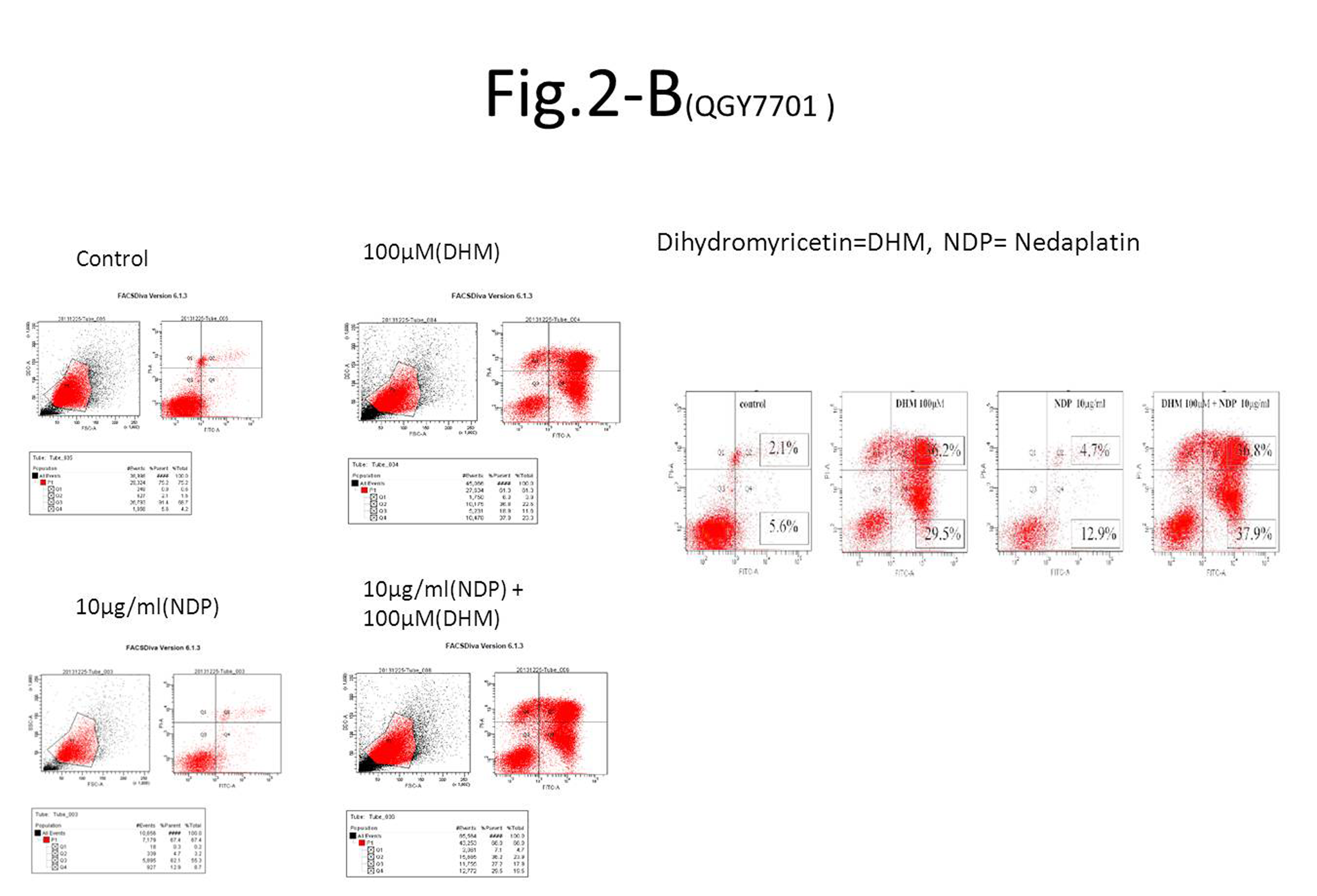

Supplement: S11 Fig — The apoptosis of cells were measured by flow cytometry analysis. (TIF) [file pone.0124994.s011.tif]

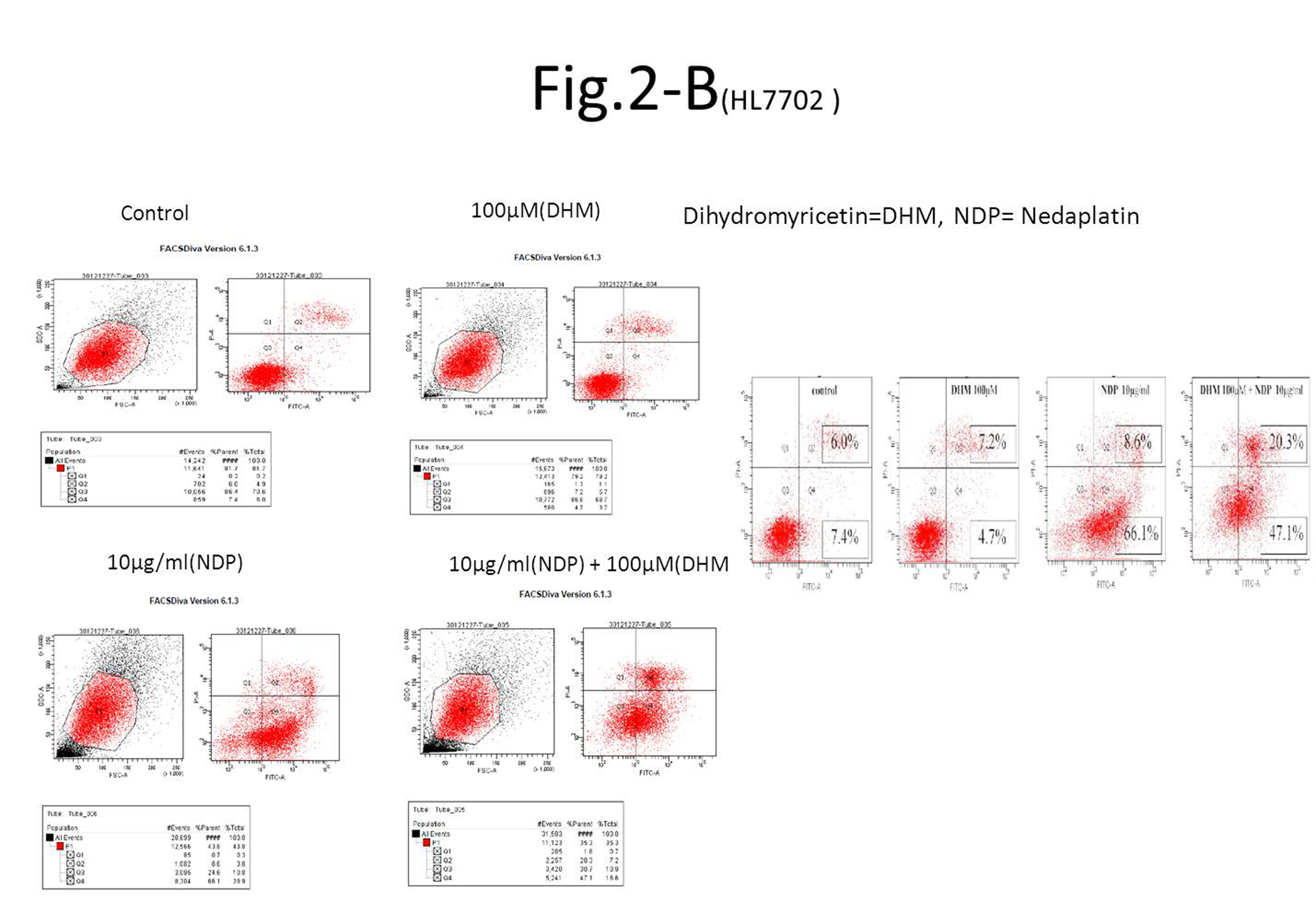

Supplement: S12 Fig — The apoptosis of cells were measured by flow cytometry analysis. (TIF) [file pone.0124994.s012.tif]

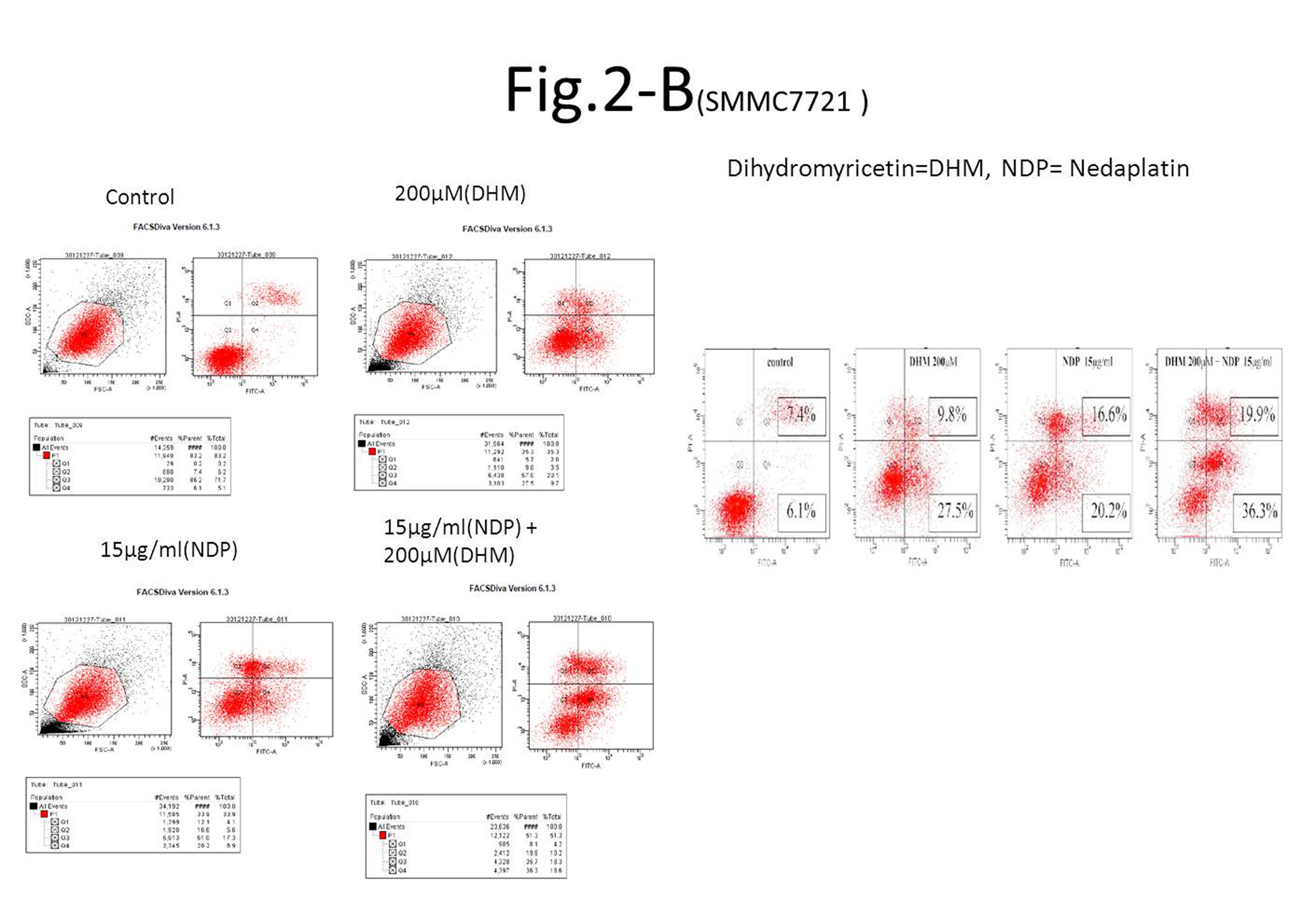

Supplement: S13 Fig — The apoptosis of were measured by flow cytometry analysis. (TIF) [file pone.0124994.s013.tif]

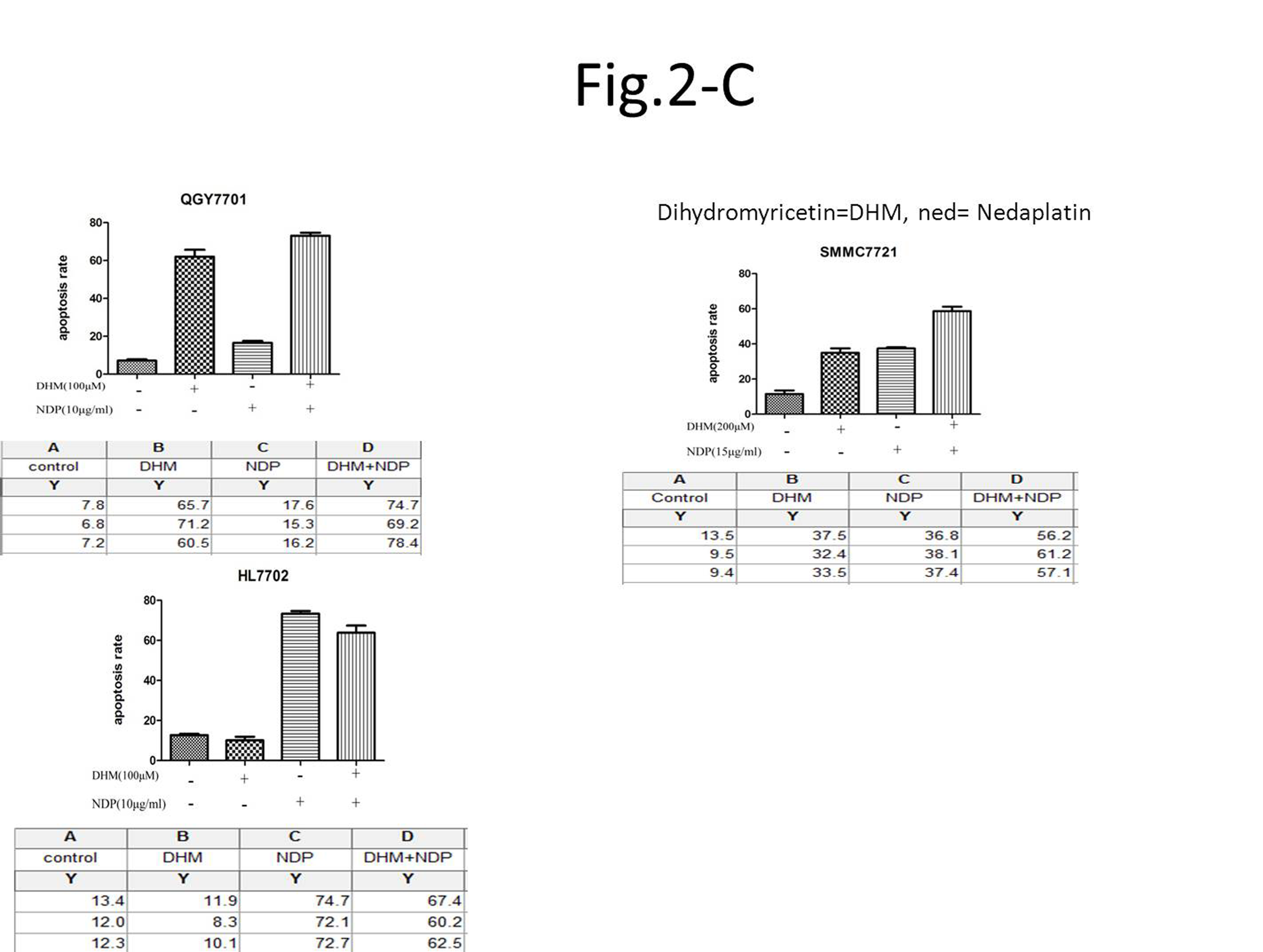

Supplement: S14 Fig — (TIF) [file pone.0124994.s014.tif]

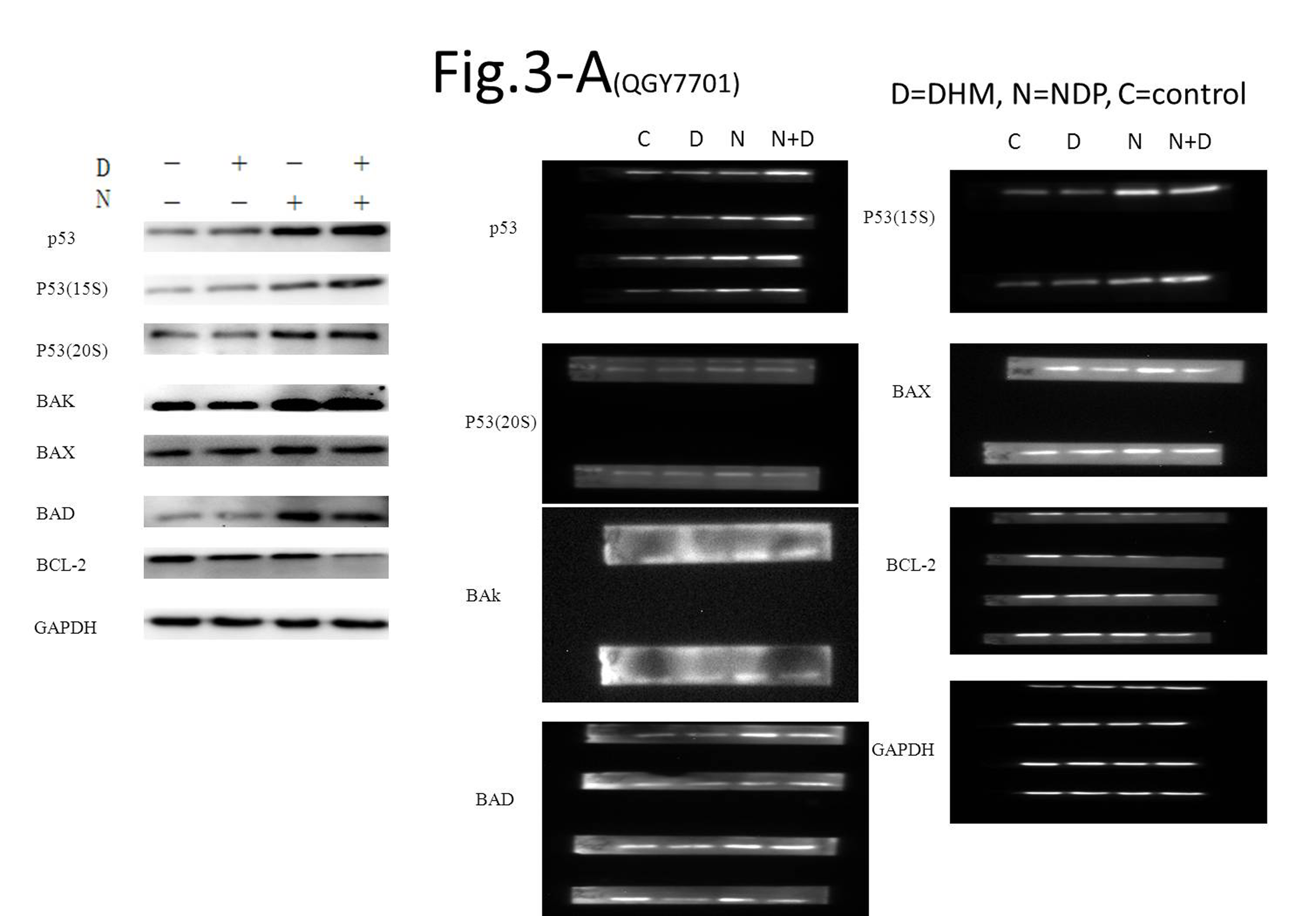

Supplement: S15 Fig — The apoptotic proteins were detected by western blot in QGY7701 cells. (TIF) [file pone.0124994.s015.tif]

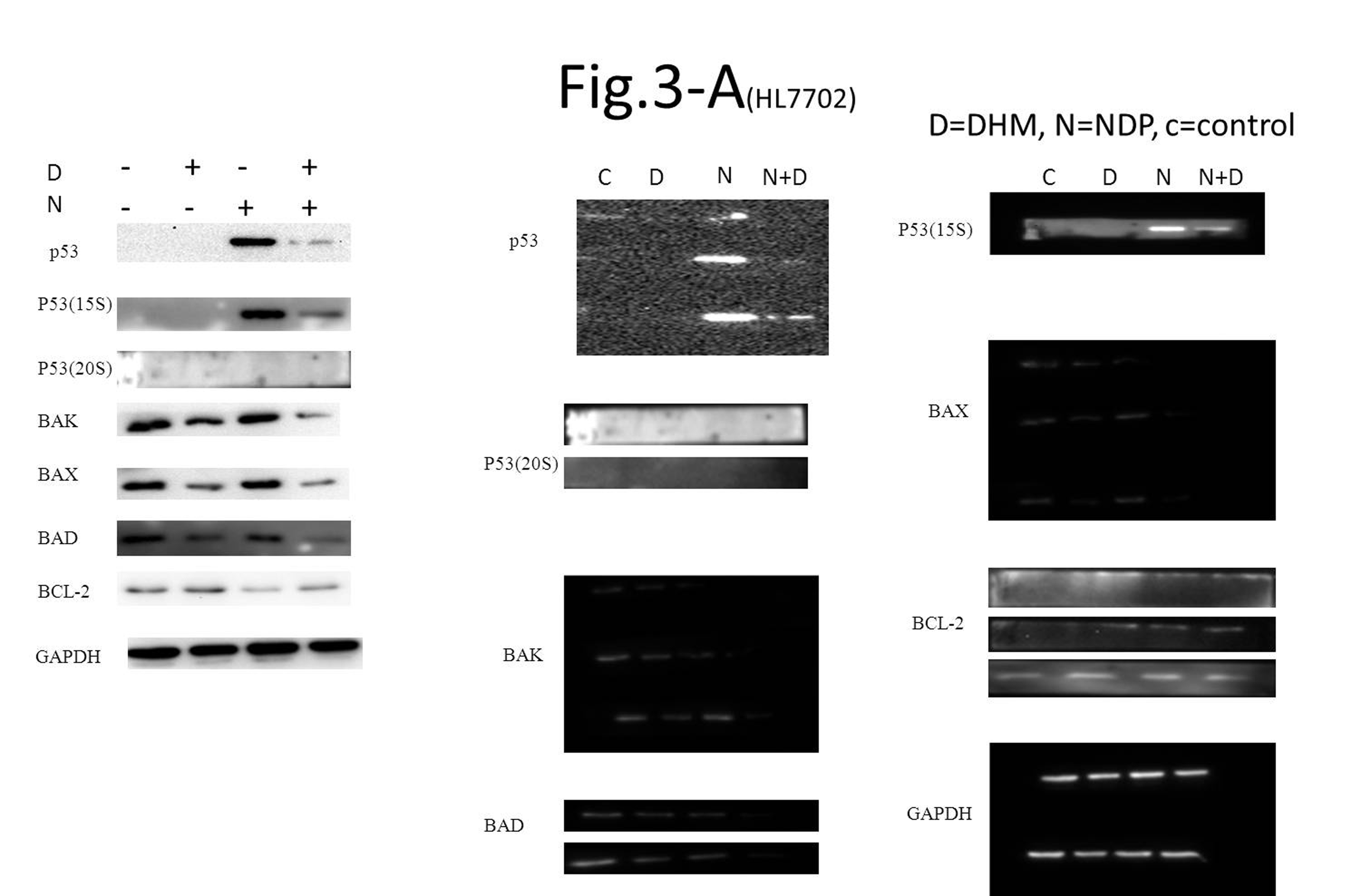

Supplement: S16 Fig — The apoptotic proteins were detected by western blot in HL7702 cells. (TIF) [file pone.0124994.s016.tif]

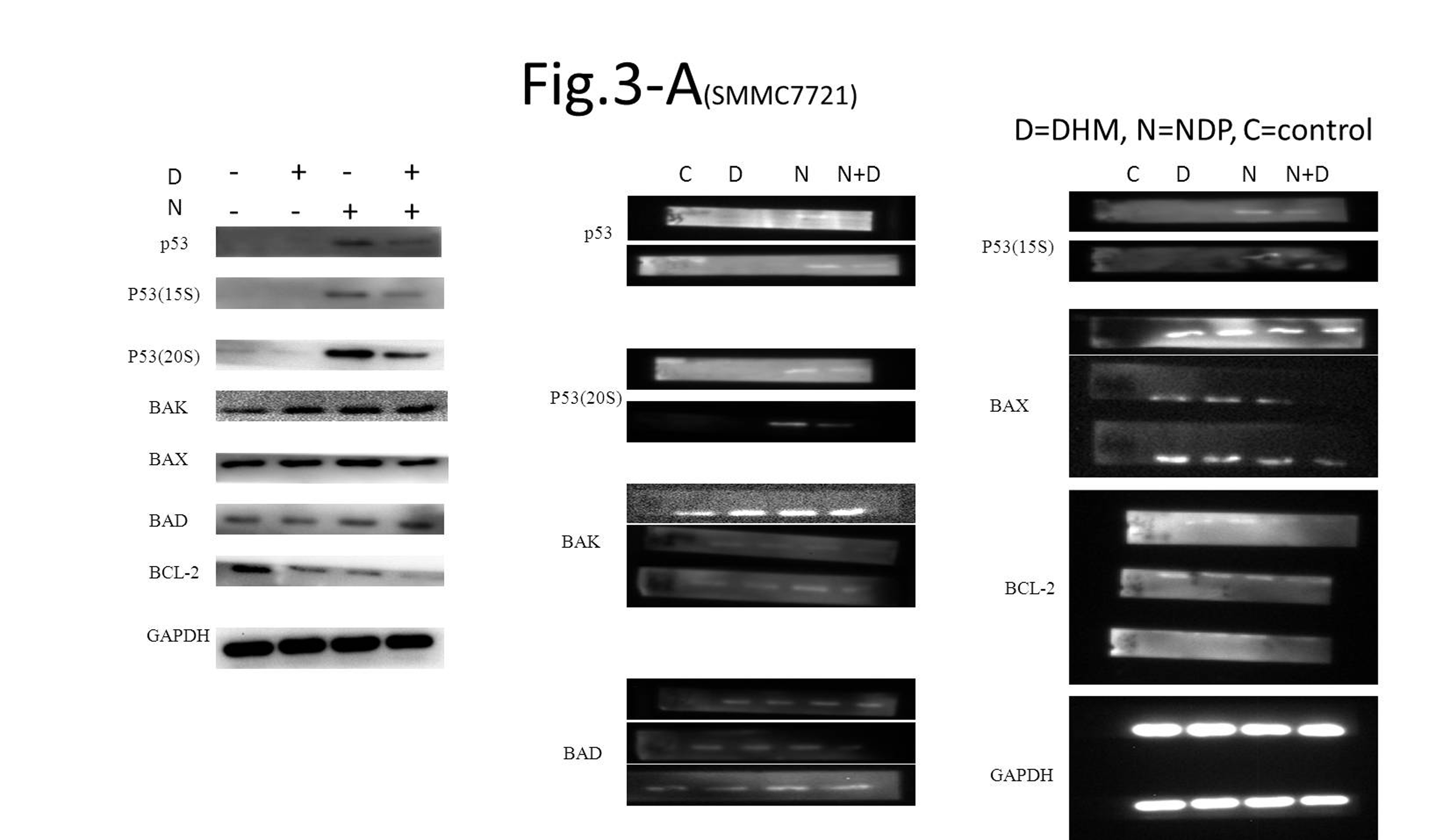

Supplement: S17 Fig — The apoptotic proteins were detected by western blot in SMMC7721 cells. (TIF) [file pone.0124994.s017.tif]

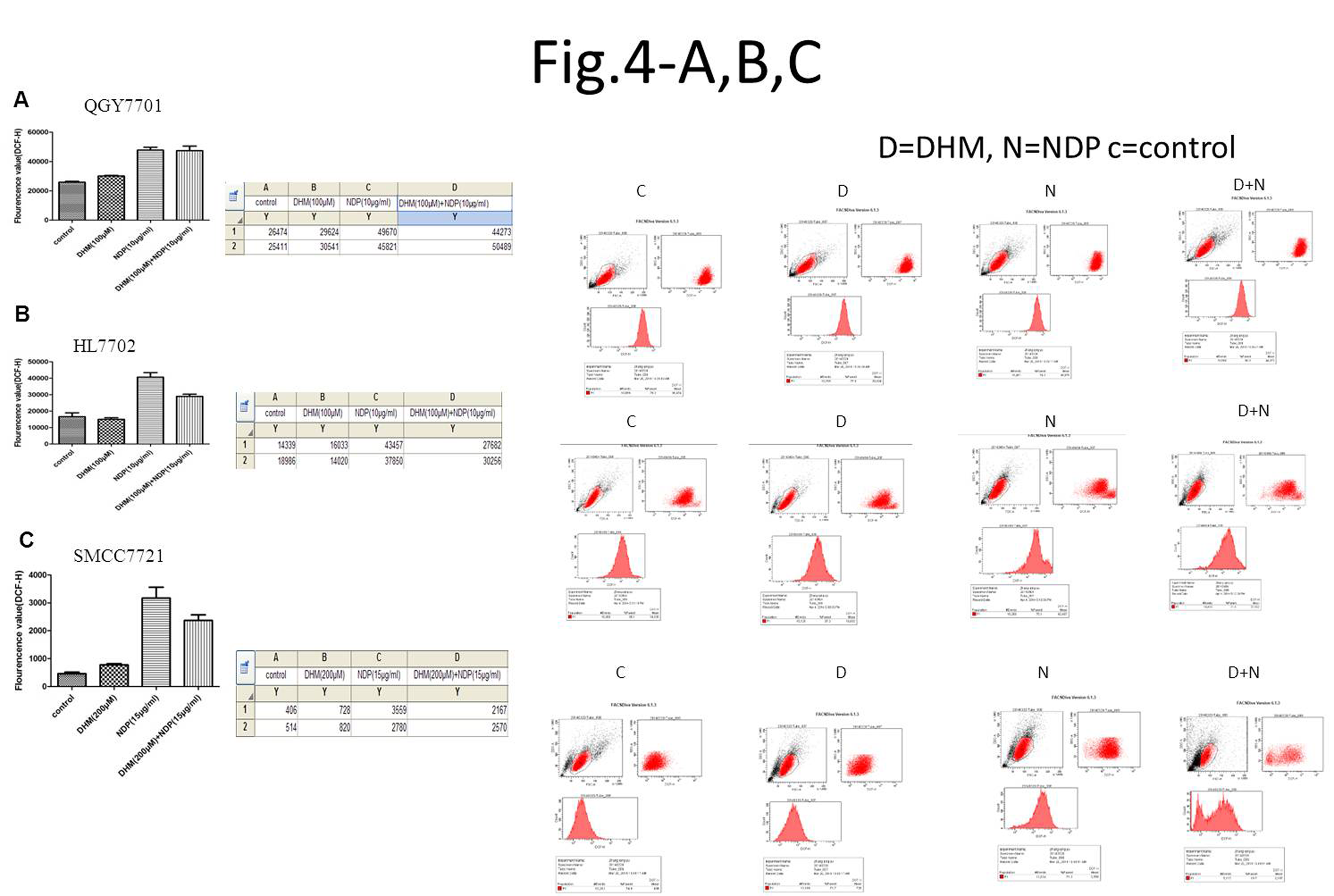

Supplement: S18 Fig — Reactive oxygen species were detected by using the DCFH assay in three cell lines (QGY7701, SMMC7721, and HL7702). (TIF) [file pone.0124994.s018.tif]

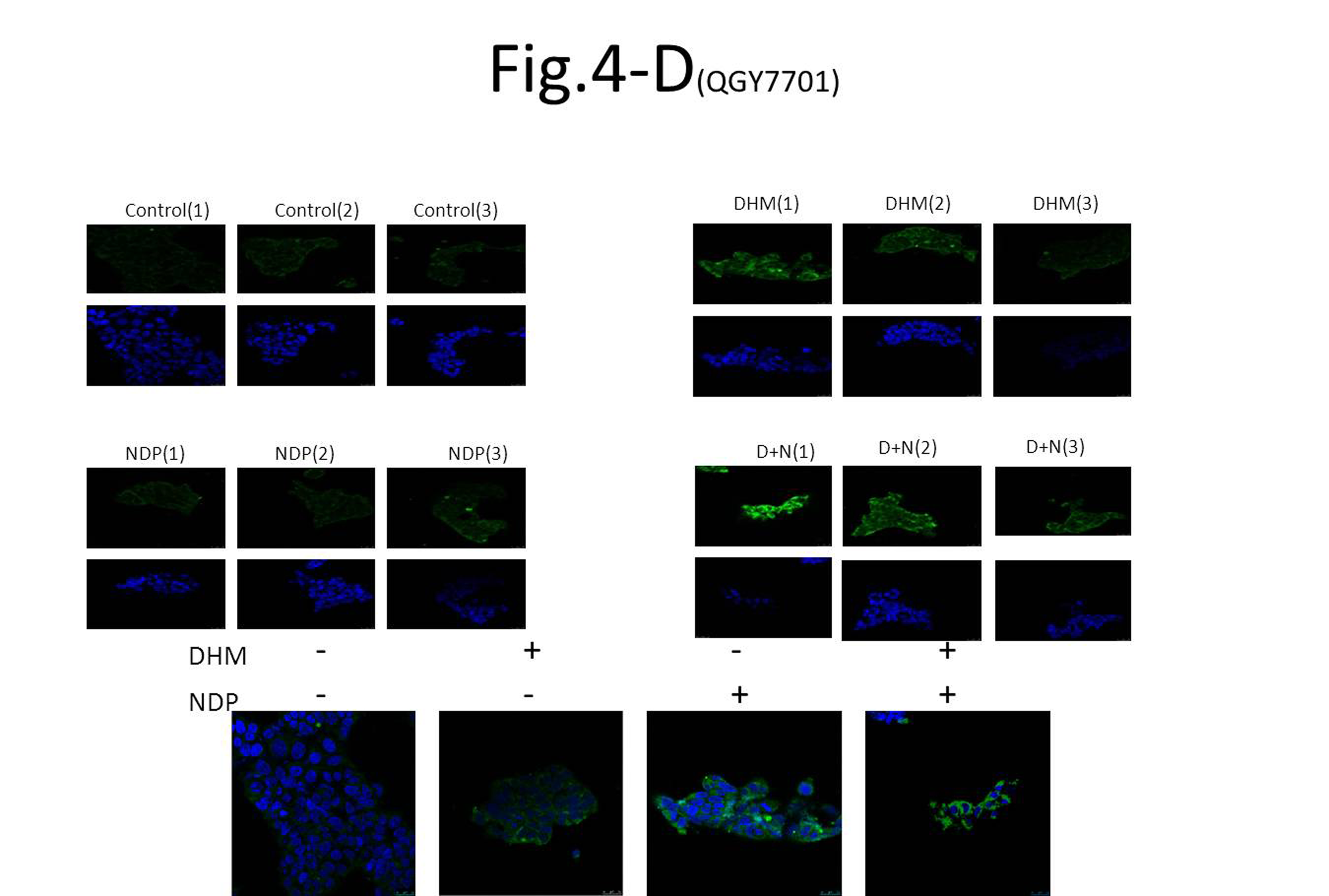

Supplement: S19 Fig — Mitochondria morphology was evaluated by mito-tracker green staining after drugs treatment in QGY7701 cells. (TIF) [file pone.0124994.s019.tif]

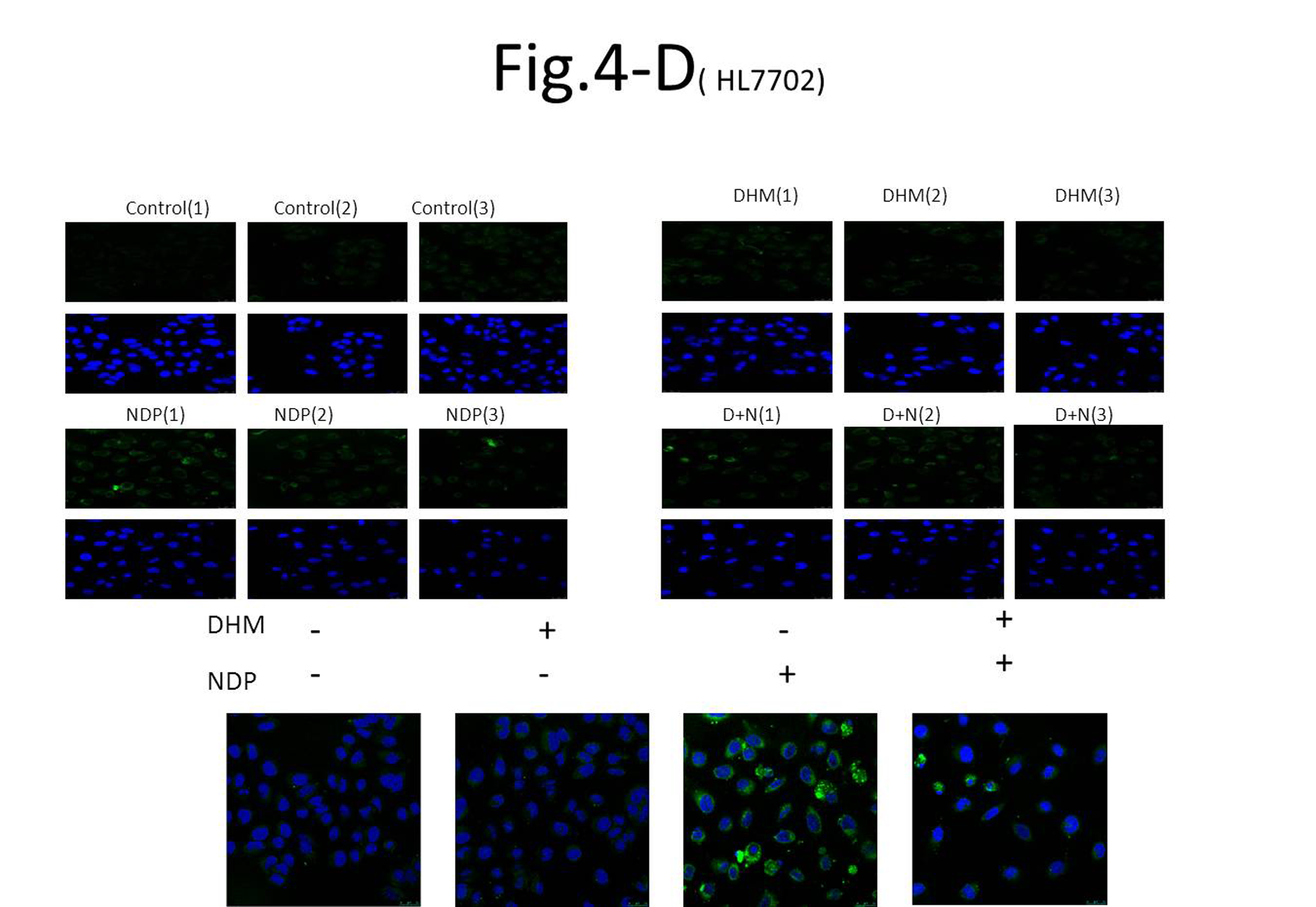

Supplement: S20 Fig — Mitochondria morphology was evaluated by mito-tracker green staining after drugs treatment in HL7702 cells. (TIF) [file pone.0124994.s020.tif]

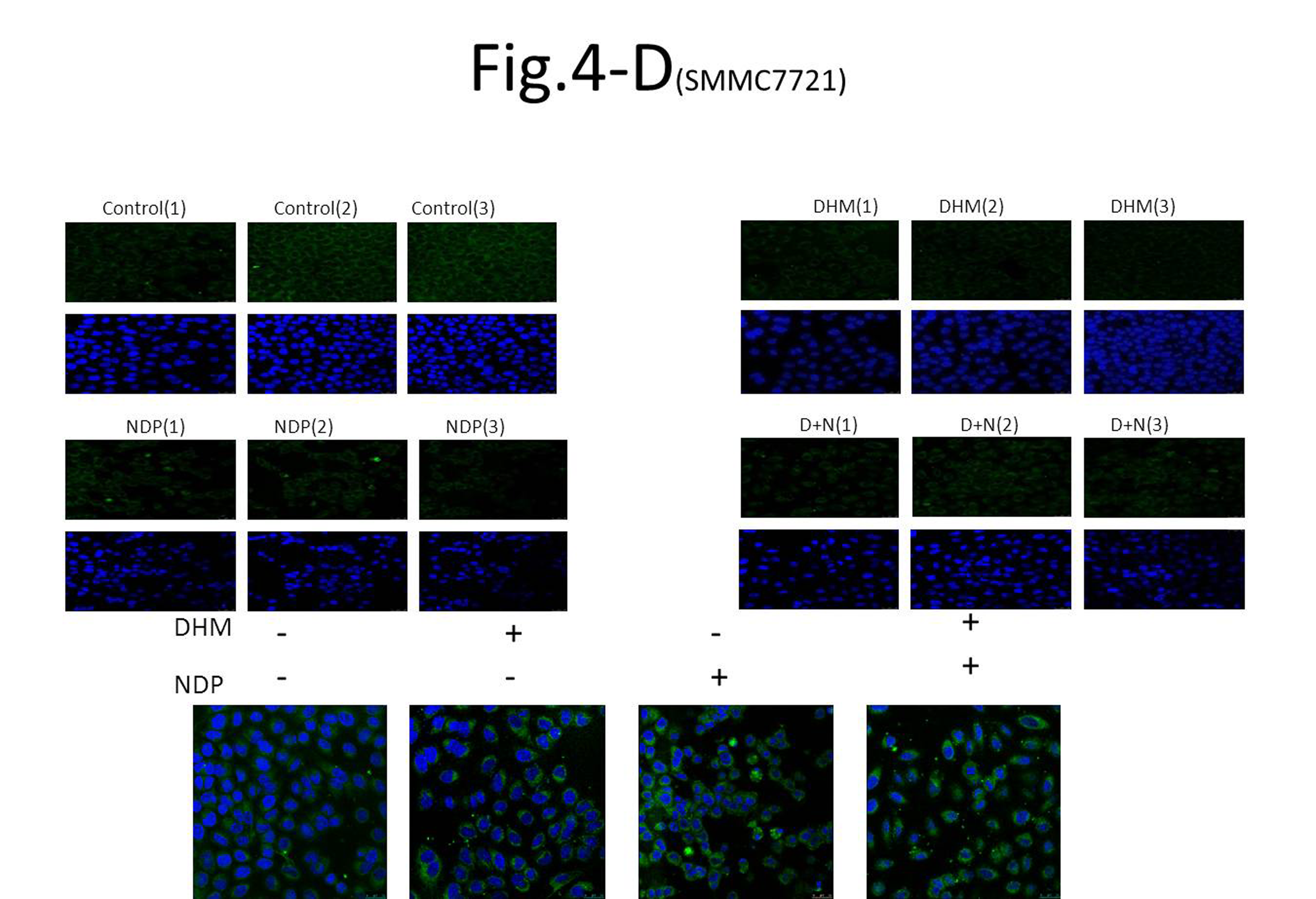

Supplement: S21 Fig — Mitochondria morphology was evaluated by mito-tracker green staining after drugs treatment in SMMC7721 cells. (TIF) [file pone.0124994.s021.tif]

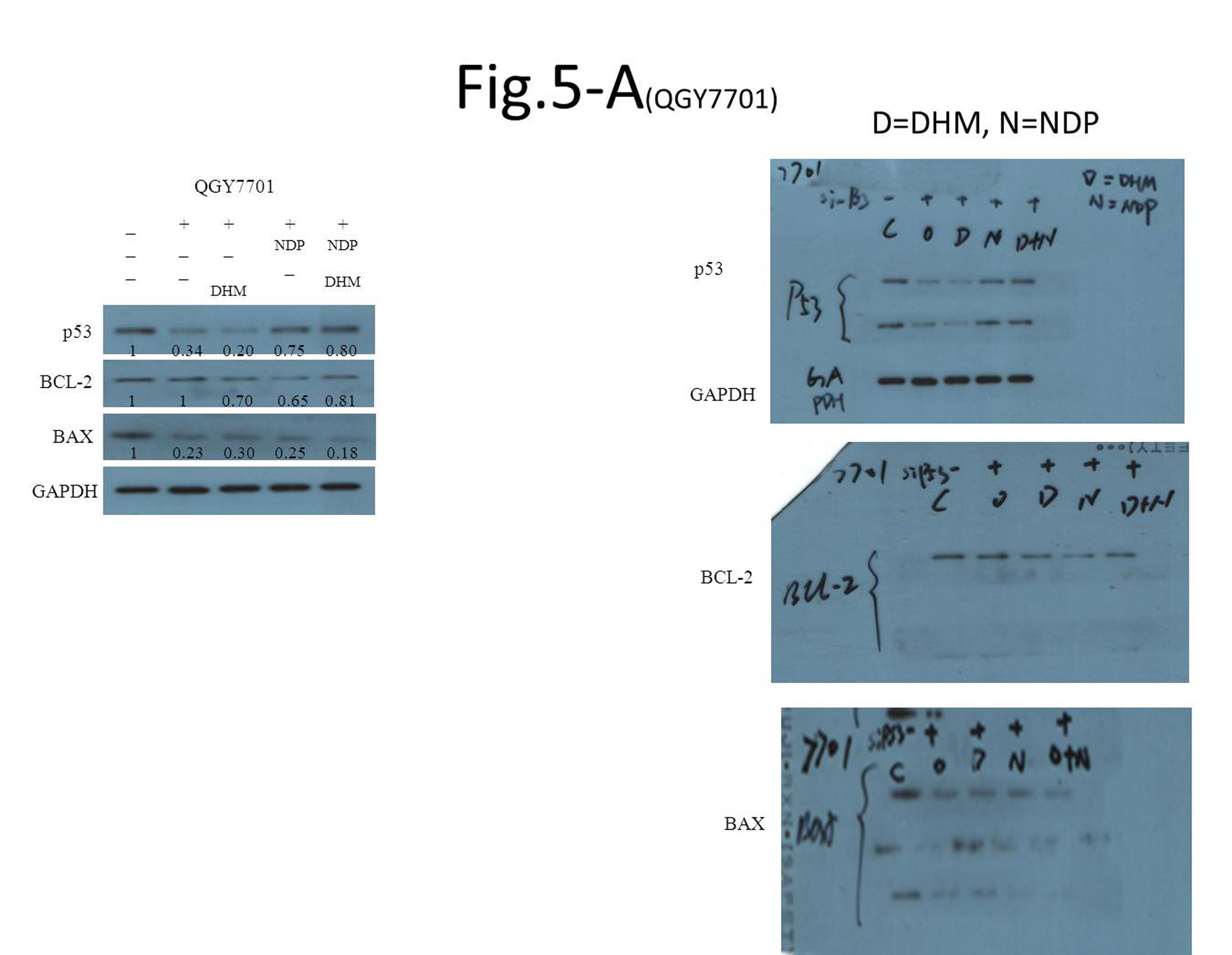

Supplement: S22 Fig — The apoptotic proteins were detected by western blot after p53 was knockdown in QGY7701 cells. (TIF) [file pone.0124994.s022.tif]

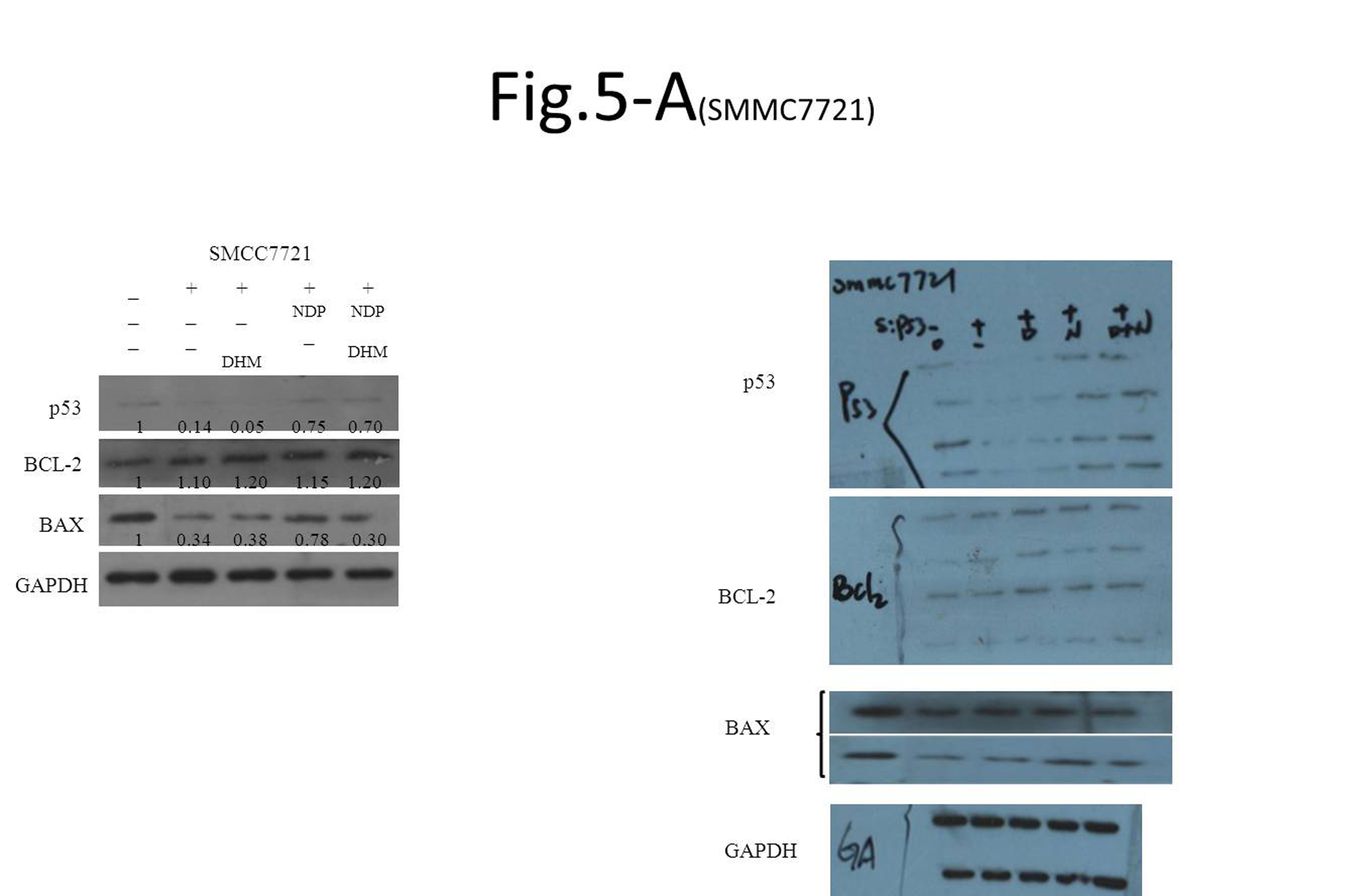

Supplement: S23 Fig — The apoptotic proteins were detected by western blot after p53 was knockdown in SMMC7721 cells. (TIF) [file pone.0124994.s023.tif]

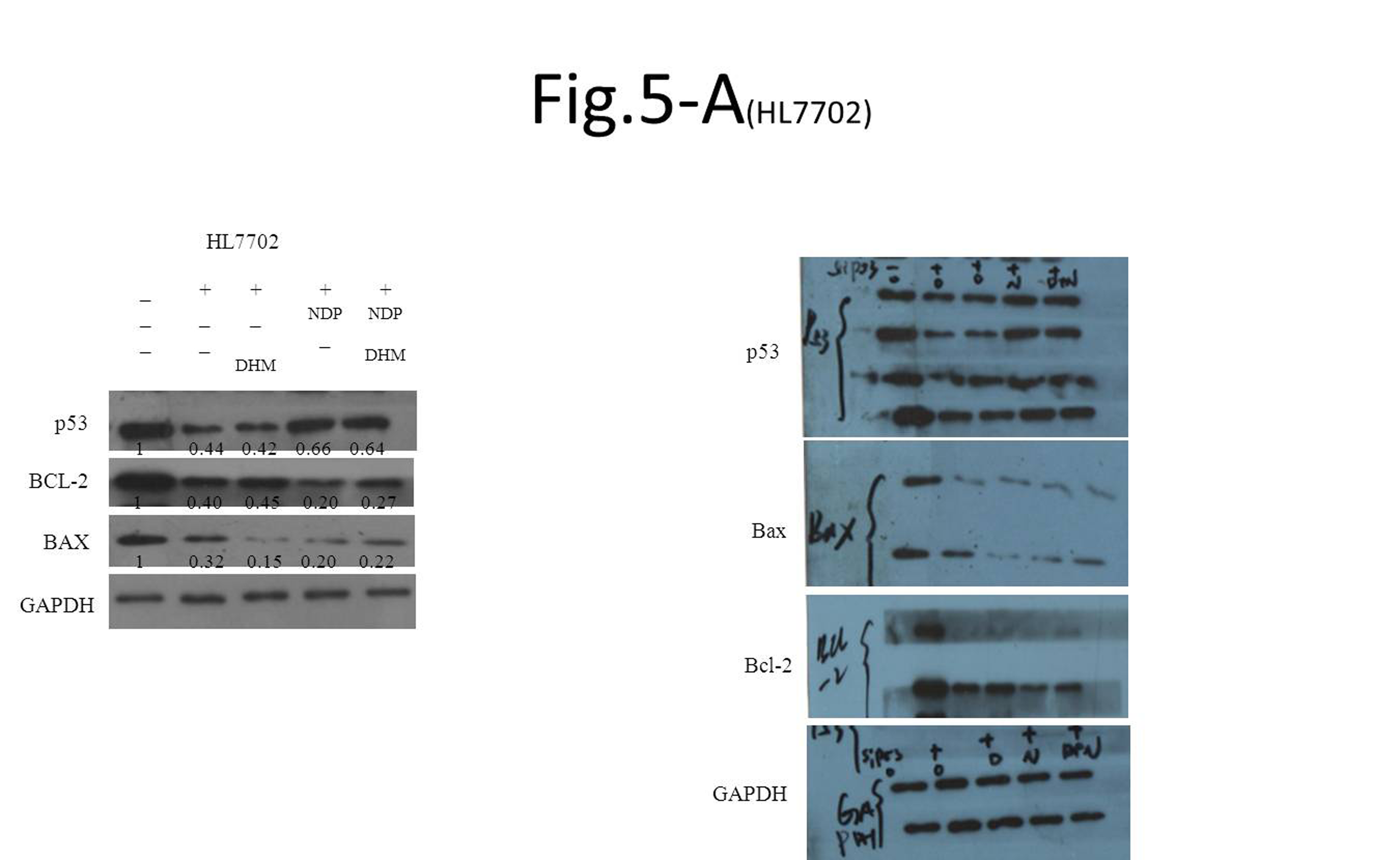

Supplement: S24 Fig — The apoptotic proteins were detected by western blot after p53 was knockdown in HL7702 cells. (TIF) [file pone.0124994.s024.tif]

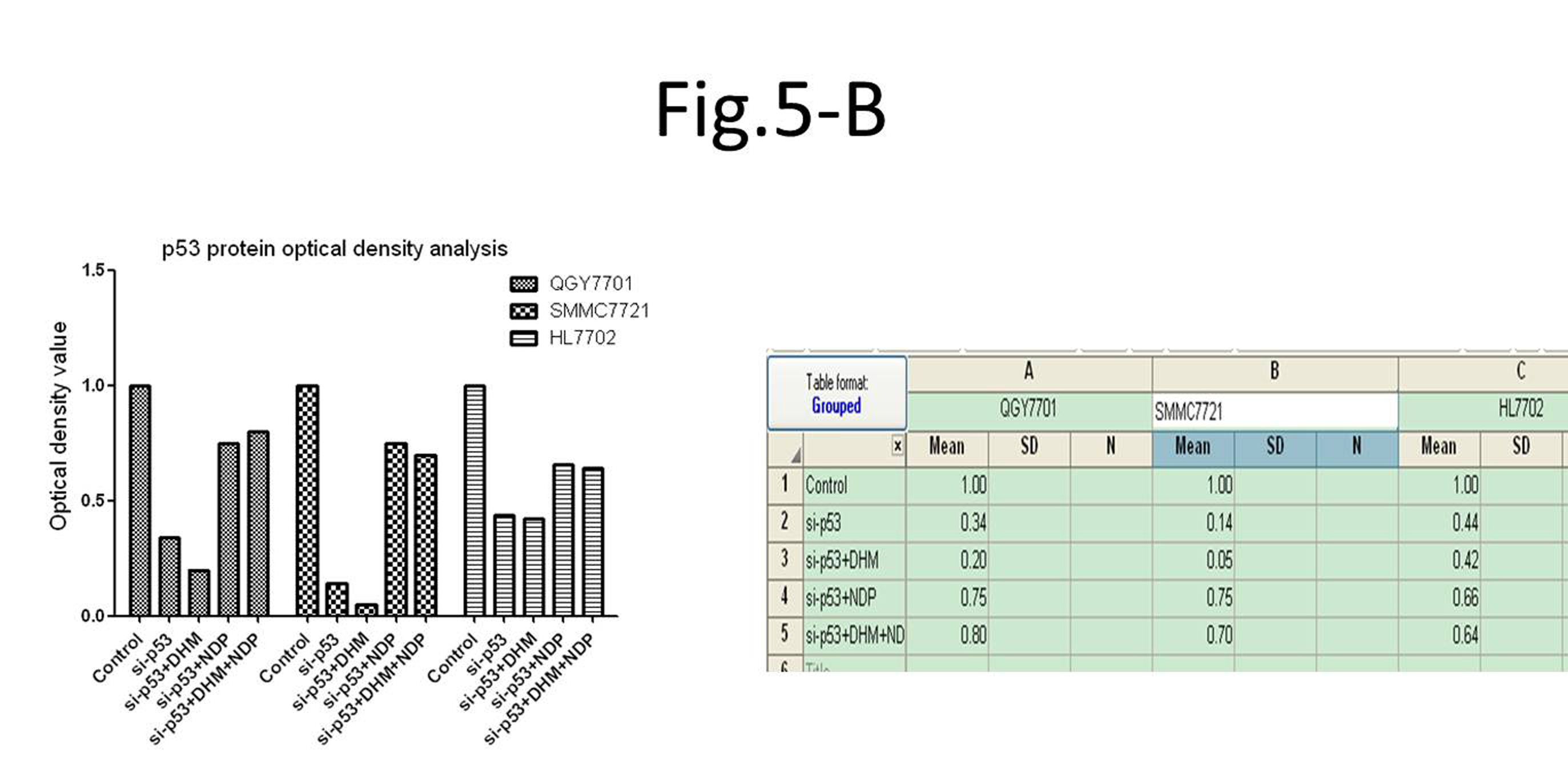

Supplement: S25 Fig — (TIF) [file pone.0124994.s025.tif]

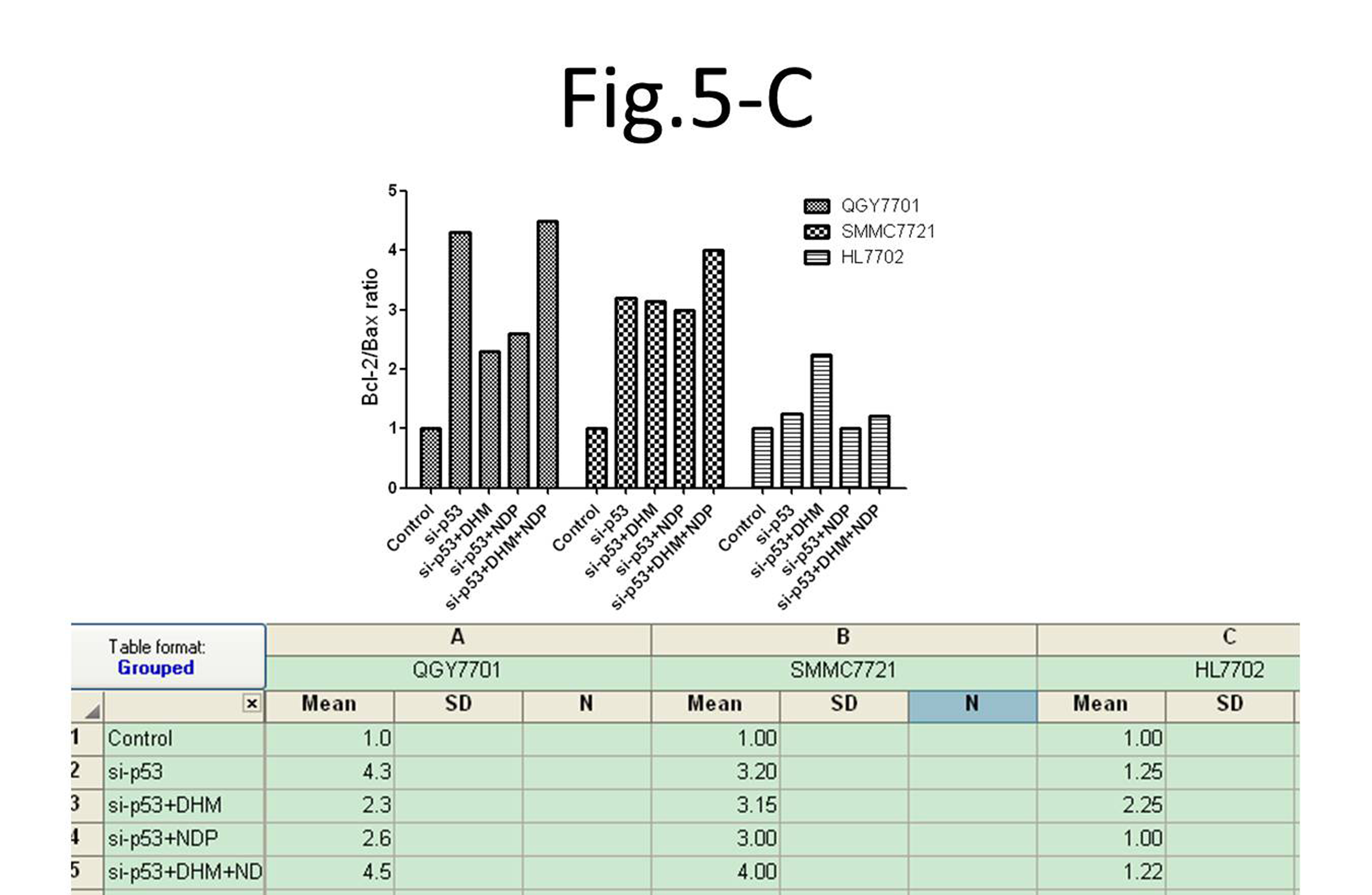

Supplement: S26 Fig — Bcl-2/Bax ratio were calculated using optical density value (TIF) [file pone.0124994.s026.tif]

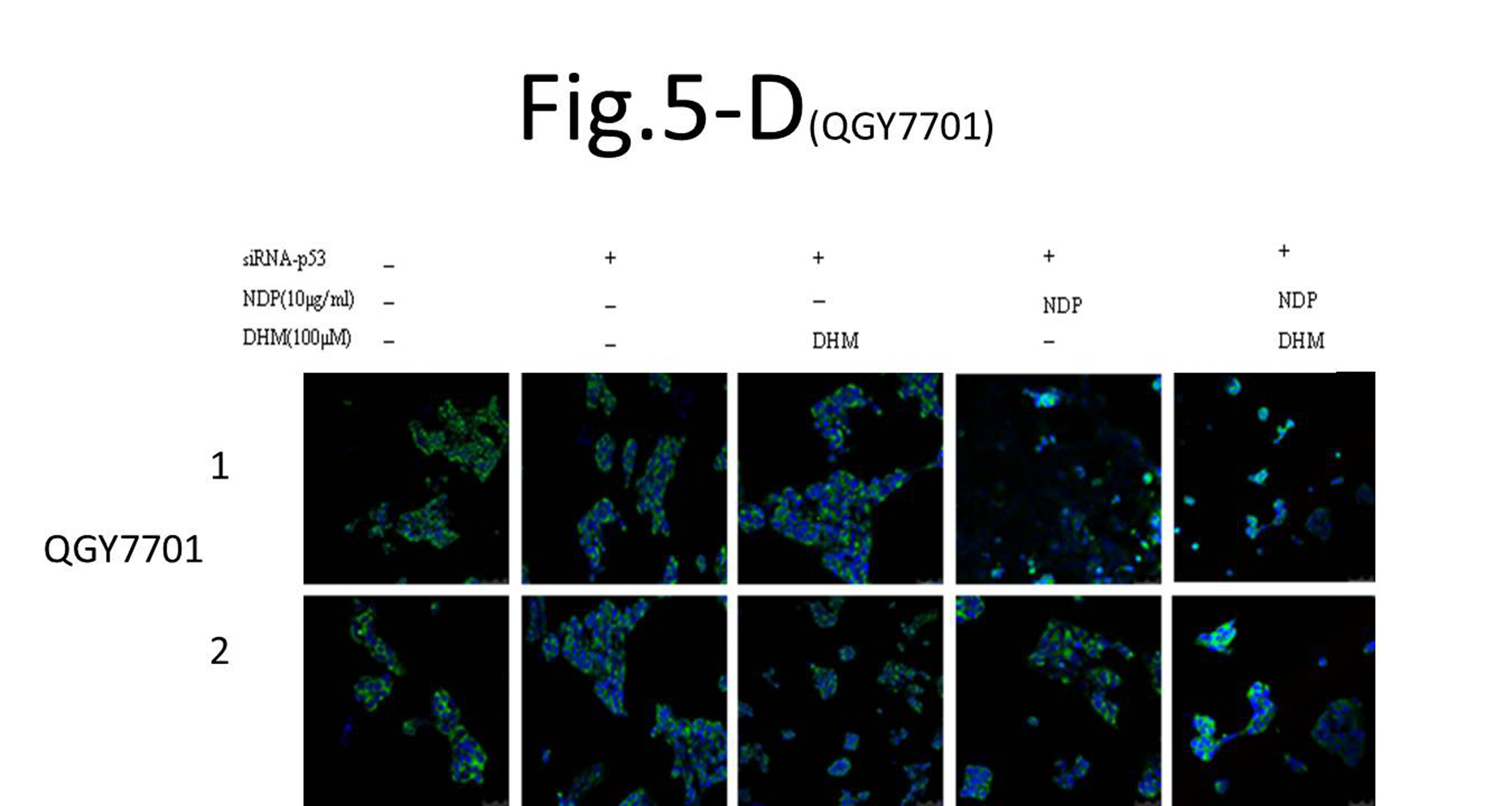

Supplement: S27 Fig — Mitochondria morphology was evaluated by mito-tracker green staining after drugs treatment in QGY7701while p53 was knockdown by siRNA transfection. (TIF) [file pone.0124994.s027.tif]

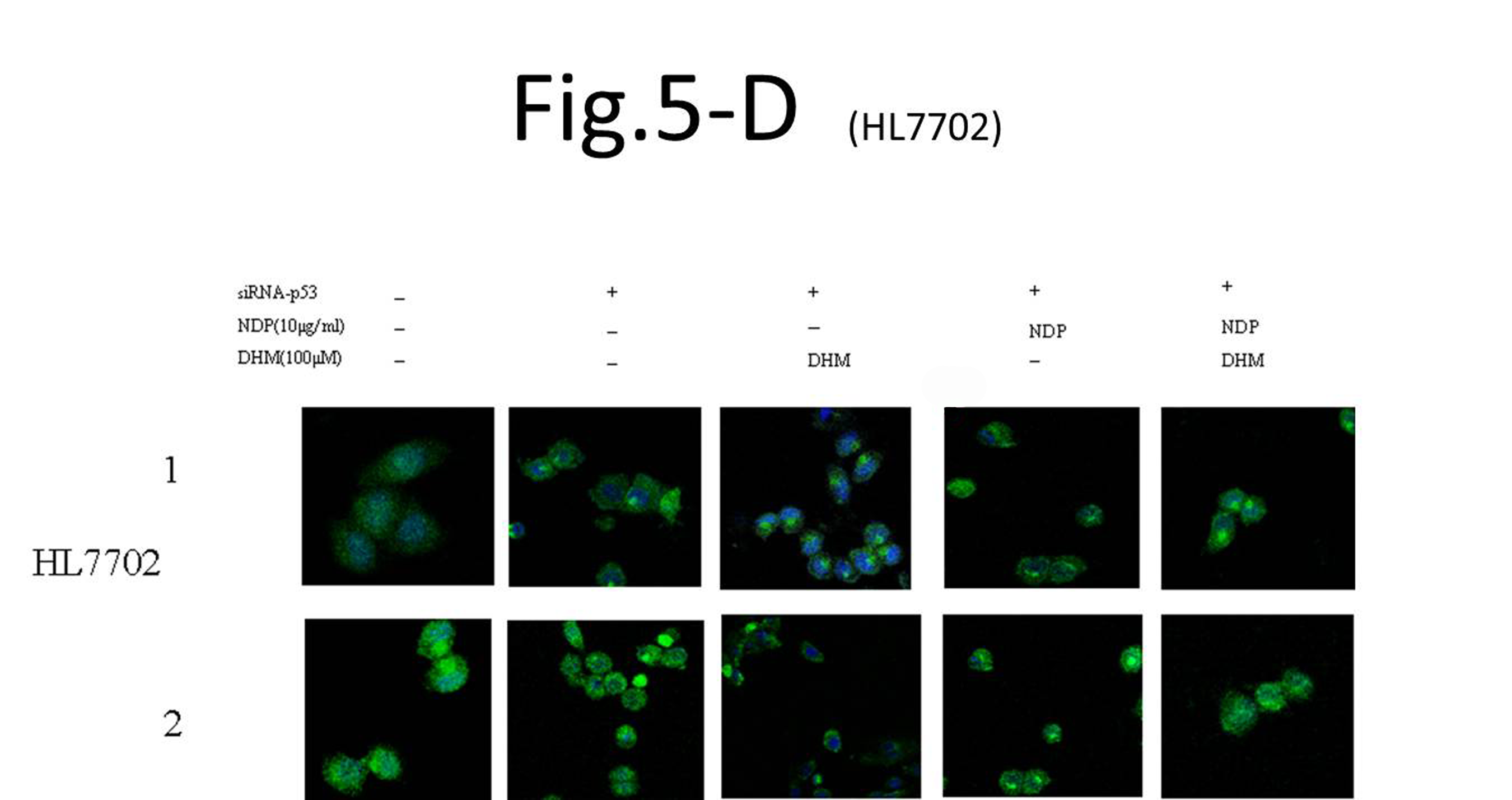

Supplement: S28 Fig — Mitochondria morphology was evaluated by mito-tracker green staining after drugs treatment in HL7702 while p53 was knockdown by siRNA transfection. (TIF) [file pone.0124994.s028.tif]

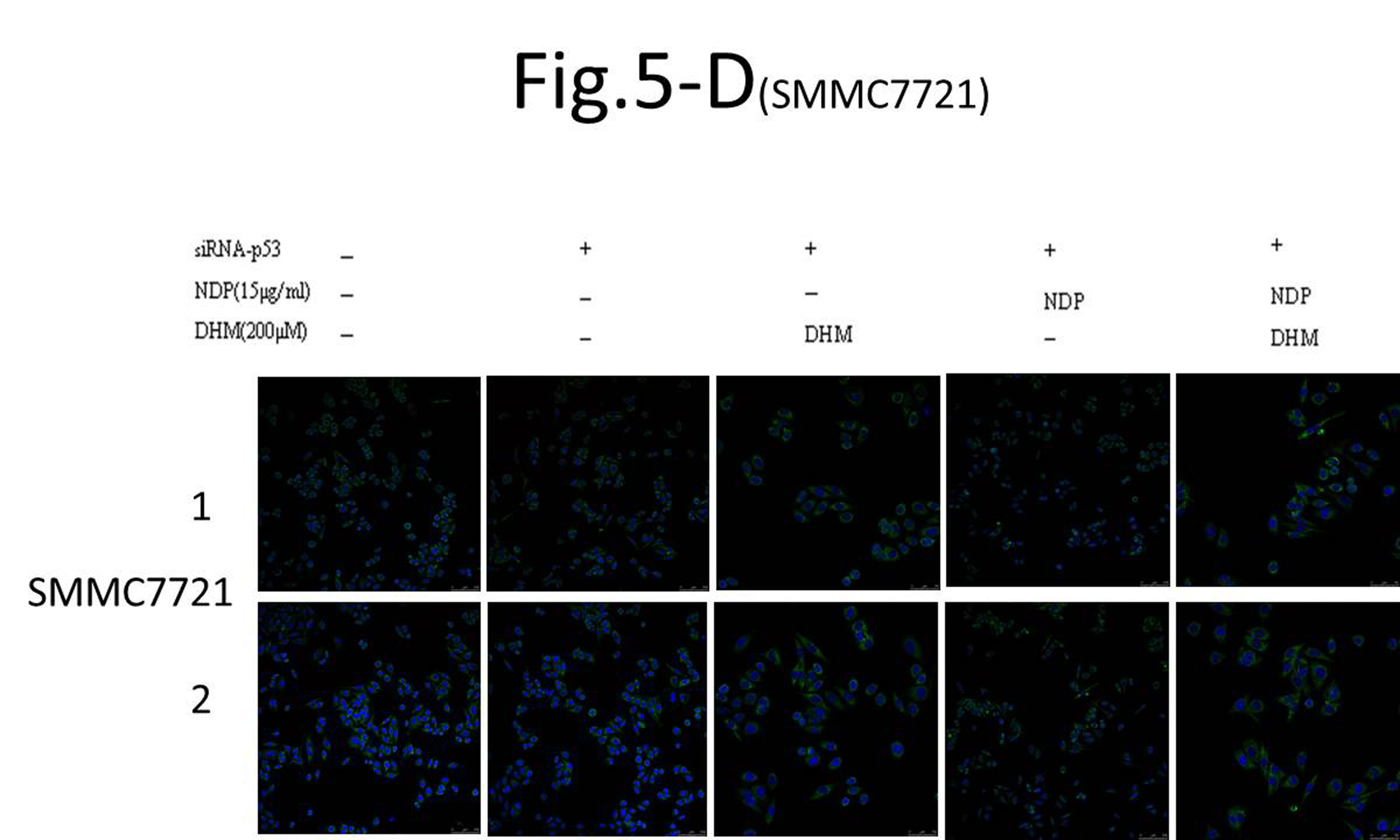

Supplement: S29 Fig — Mitochondria morphology was evaluated by mito-tracker green staining after drugs treatment in SMMC7721 while p53 was knockdown by siRNA transfection. (TIF) [file pone.0124994.s029.tif]
